# *Bombyx mori* β1,4-*N*-acetylgalactosaminyltransferase possesses relaxed donor substrate specificity in *N*-glycan synthesis

**DOI:** 10.1038/s41598-021-84771-z

**Published:** 2021-03-09

**Authors:** Hiroyuki Kajiura, Ryousuke Miyauchi, Akemi Kakudo, Takao Ohashi, Ryo Misaki, Kazuhito Fujiyama

**Affiliations:** International Center for Biotechnology, Osaka University, 2-1 Yamada-oka, Suita-shi, Osaka, 565-0871 Japan

**Keywords:** Glycobiology, Post-translational modifications

## Abstract

*N*-Glycosylation is one of the most important post-translational protein modifications in eukaryotic cells. Although more than 200 *N*-glycogenes contributing to *N*-glycan biosynthesis have been identified and characterized, the information on insect *N*-glycosylation is still limited. Here, focusing on insect *N*-glycosylation, we characterized *Bombyx mori N*-acetylgalactosaminyltransferase (BmGalNAcT) participating in complex *N*-glycan biosynthesis in mammals. BmGalNAcT localized at the Golgi and was ubiquitously expressed in every organ and in the developmental stage of the middle silk gland of fifth instar larvae. Analysis of recombinant BmGalNAcT expressed in Sf9 cells showed that BmGalNAcT transferred GalNAc to non-reducing terminals of GlcNAcβ1,2-R with β1,4-linkage. In addition, BmGalNAcT mediated transfer of galactose and *N*-acetylglucosamine residues but not transfer of either glucose or glucuronic acid from the UDP-sugar donor substrate to the *N*-glycan. Despite this tri-functional sugar transfer activity, however, most of the endogenous glycoproteins of insect cells were present without GalNAc, Gal, or GlcNAc residues at the non-reducing terminal of β1,2-GlcNAc residue(s). Moreover, overexpression of *BmGalNAcT* in insect cells had no effect on *N*-acetylgalactosaminylation, galactosylation, or *N*-acetylglucosaminylation of the major *N*-glycan during biosynthesis. These results suggested that *B. mori* has a novel multifunctional glycosyltransferase, but the *N*-glycosylation is highly and strictly regulated by the endogenous *N*-glycosylation machineries.

## Introduction

The final step of gene expression involves the functional protein biosynthesis. In higher eukaryotic cells, most proteins are hardly functional in polypeptide chain and further require post-translational modifications to express their diversity of physiological and biological activities. Among the post-translational modifications, *N*-glycosylation, the addition of sugar residues to asparagine residues in the context of N-X-S/T (where X is any amino acid except proline) on the peptide backbone, plays important roles in protein folding, assembly, and control of the metabolic rates of proteins by protecting proteins from proteolysis or antigenic recognition^1–4^. *N*-Glycans are synthesized by the actions of highly conserved glycosyltransferases (GT) and glycosylhydrolases in the endoplasmic reticulum (ER) and Golgi. The structure of *N*-glycan biosynthesized in the ER is universal, but GTs functioning in *medial*- to *trans*-Golgi have species specificities, resulting in the structural diversity of species-specific *N*-glycans.

The details of *N*-glycosylation in insects, especially within Golgi, have been controversial. The distinctive *N*-glycan structure referred to as insect type is 3 mannoses (Man3) with α1,3- and α1,6-fucose residues (M3FF). Thus, insects have active α1,3-fucosyltransferase (FUCT) and α1,6-FUCT, which are particularly distributed in plants and mammals, respectively^5–8^. However, except in honeybees, M3FF is not general and is hardly detected in total *N*-glycan^9^, even though Man2s and/or Man3s with α1,3-fucose (Fuc) or α1,6-Fuc are abundant, indicating that α1,3-FUCT and α1,6-FUCT compete for an identical acceptor substrate^10,11^. Another curious feature of insect GT is α2,6-sialyltransferase (α2,6-ST). Drosophila and silkworm possess the α*2,6-ST* gene, but the reaction product of endogenous sialylated *N*-glycans has not been detected except for Drosophila embryos, suggesting that insect α2,6-STs are almost non-functional enzymes in vivo^12,13^. Remarkably, even though insect α2,6-STs are inactive in vivo, insect α2,6-STs show an in vitro substrate preference different from that of mammals; insect α2,6-STs prefer β-linked *N*-acetylgalactosamine (GalNAc) residue(s) to galactose (Gal) residue at the non-reducing terminus, and efficiently transfer *N*-acetylneuraminic acid (NeuAc) to arylglycosides substrates rather than *N*-glycans^13–15^. These results suggested that insects might have specific GT(s), i.e., insect β1,4-*N*-acetylgalactosaminyltransferase(s) (GalNAcT(s)) that are more suitable for sialylation of *N*-glycans than β1,4- and β1,3-galactosyltransferase (GALT). Actually, endogenous β1,4-*N*-acetylgalactosaminylated *N*-glycans have been detected in insects, such as *Drosophila*, mosquito, honeybees, and lepidopteran larvae^16–21^ and some insect GalNAcTs were identified^22–24^, suggesting that insects have a potential for synthesizing LacdiNAc structures on *N*-glycans.

*N*-Acetylgalactosaminyltransferases are divided into several families. One of the major GalNAcTs contributing to *N*-glycan biosynthesis are categorized as a Glycosyltransferase Family 7 (GT7) proteins in the Carbohydrate-Active enZYmes database and are mainly distributed in invertebrates, unlike β1,4-GALTs, which are distributed in vertebrates. The other well-known GalNAcTs are UDP-*N*-acetyl-α-d-galactosamine:polypeptide *N*-acetylgalactosaminyltransferases (αppGalNAcTs), which are categorized as GT27 proteins responsible for the biosynthesis of mucin-type *O*-glycans^25^. GalNAcTs and αppGalNAcTs have different substrate specificities and catalyze the GalNAc transfer to glycan to produce a LacdiNAc structure or a polypeptide acceptor from an activated sugar, UDP-GalNAc, respectively. The protein structure and sequences of catalytic domains lead us to propose that GalNAcTs and αppGalNAcTs were emerged from a common ancestral gene^26^. Interestingly, a single amino acid residue on GalNAcTs, Ile/Leu, is a key determinant affecting the donor sugar specificity; GalNAcT converts to β1,4-GALT^27^. In fact, GalNAcTs possess weak Gal transferase activity^24,27^, supporting the notion that GalNAcTs could play the same role as β1,4-GALTs. Thus, the evolutional substitution of Ile/Leu on GalNAcTs resulting in β1,4-GALT emergence, is indispensable for changing the glycosylation of glycoproteins and glycolipids in vertebrates. In other words, similar to β1,4-GALTs in vertebrates^28–30^, GalNAcTs might play critical role(s) in physiological and/or biological functions in invertebrates. Indeed, a mutation in Drosophila GalNAcT resulted in different behavioral phenotypes in adult flies, and the neural and muscle phenotypes in larvae^23,31^ and *Caenorhabditis elegans* GalNAcT mutant, *ngat-1*, showed temperature sensitivity and defects in cell migration^32^. These facts demonstrate that GalNAcTs play important functions in vivo and might explain their presence*.* However, information on the function of GalNAcTs and the LacdiNAc structure on *N*-glycans in vivo is still limited. Therefore, additional information on GalNAcTs and their in vivo function is needed. Information on the genome sequence of the silkworm, *Bombyx mori*, has been made available^33^. *B. mori* is an invertebrate, and thus it is likely to possess GalNAcT rather than β1,4-GALT^23,24,26^. The above facts should help us to identify a *B. mori* GalNAcT homologue and to elucidate the physiological and developmental roles of LacdiNAc structure in insects. Moreover, the recent finding that α2,6-ST is present in *B. mori* also supports the idea that *B. mori* possesses an active GalNAcT like that in Drosophila possessing both GalNAcT and α2,6-ST. Recently, *B. mori* GalNAcT was identified and shown to be enzymatically active^34^. However, its donor substrate preference, its subcellular localization and contribution to in vivo* N*-glycan biosynthesis were not extensively addressed.

Here, focusing on the insect GalNAcT, we characterized *B. mori* GalNAcT (BmGalNAcT). *BmGalNAcT* encoded a protein of 420 amino acids and was localized in the Golgi as *N*-glycoproteins. BmGalNAcT transferred GalNAc residues to the various acceptor substrates that contained terminal *N*-acetylglucosamine (GlcNAc) residue(s) with β1,4-linkage. BmGalNAcT transferred Gal residues as expected, but it also transferred GlcNAc residues. Although BmGalNAcT was successfully expressed in insect cells, the enhancement of LacdiNAc formation on major *N*-glycans in vivo was not observed despite in vitro activity; this suggests some additional requirement for GalNAc transfer during *N*-glycan processing.

## Results

### GALT family topology of the putative *B. mori N*-acetylgalactosaminyltransferase

In insects, β1,4-GALT orthologs GalNAcT from Drosophila, *Trichoplusia ni*, *Spodoptera frugiperda*, and *Mamestra brassicae* have been identified and characterized^22–24^. Based on the amino acid sequences of human β1,4-GALT, a database search using the KAIKOBase (http://sgp.dna.affrc.go.jp/KAIKObase/) led to the identification of a putative *B. mori* GalNAcT on the genome. The sequence was encoded on chromosome 3 and assigned as BGIBMGA007485 in KAIKOBase. The *B. mori* GalNAcT (BmGalNAcT) is a protein of 420 amino acids with a calculated molecular mass of 48.5 kDa. BmGalNAcT possessed 10 potential *N*-glycosylation sites. BmGalNAcT had a hydrophobic transmembrane sequence at the N-terminus, suggesting that BmGalNAcT localizes at the ER or Golgi as a type II transmembrane protein. The amino acid alignments with *Drosophila melanogaster* GalNAcTs (DmGalNAcTA and DmGalNAcTB) and *Trichoplusia ni* GalNAcT (TnGalNAcT) are shown (Supplementary Fig. S1). Though the N-termini of the sequences were dissimilar to each other, the C-termini of the sequences had higher similarities. This agreed with the idea that the catalytic domain of glycosyltransferases categorized into the same family should be conserved. In fact, BmGalNAcT also had the highly conserved sequences including the catalytic domain, binding sites of the donor and acceptor substrates and metal ions, which were observed at the C-terminal regions of GT 7-categolized GALT family proteins and responsible for showing the GALT activity^27,35,36^. Interestingly, BmGalNAcT had a putative long stem region, Leu37 to Gly164, which agreed well with the characteristics of β1,4-GALT^37^. In addition, most of the putative *N*-glycosylation sites, 8 of 10, were positioned on the stem region, indicating that the *N*-glycan on BmGalNAcT had little effect on its activity.

Ramakrishnan et al. showed that Ile289 of DmGalNAcT was an important factor for GalNAcT transfer activity^26,27^. The substitution of Ile289 to Tyr resulted in showing major β1,4-galactose transfer activity, and therefore the site is a key determinant and a hallmark of GALT family protein for sugar transfer. The amino acid of the corresponding site, Ile289 of DmGalNAcT, was Ile in BmGalNAcT (Ile310). In addition, the phylogenetic analysis using mammalian GALTs and invertebrate GalNAcTs suggested that BmGalNAcT transfers *N*-acetylgalactosamine rather than galactose to *N*-glycan (Supplementary Fig. S2).

### Expression and localization of BmGalNAcT

In order to confirm the specificities of *BmGalNAcT* expression in *B. mori*, the expression levels were examined by quantitative RT-PCR using various kinds of cDNAs isolated from different organs and developmental stages of the middle silk gland (MSG) in 5th instar larvae (Supplementary Fig. S3 and the source data for the figure is provided in Supplementary Data 1). The expression level was different to a varying degree and showed organ-dependent expression, but *BmGalNAcT* was constitutively expressed among all organs and in the developmental stage of MSG. These results suggested that if the BmGalNAcT was active, the reaction product, the LacdiNAc structure, was detected in *N*-glycoprotein(s), *O*-glycoprotein(s) or glycolipid(s).

**Figure 1:**
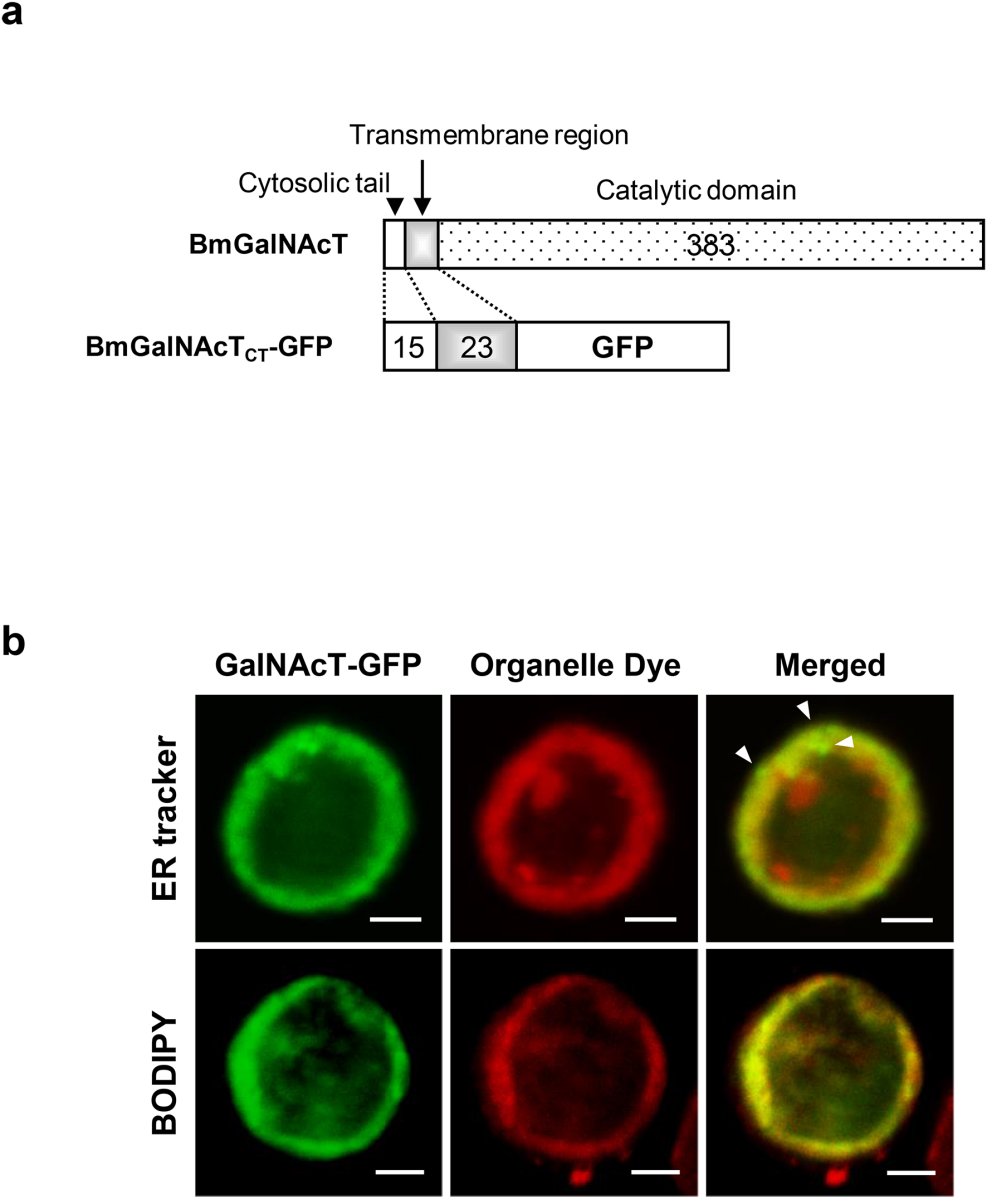
Subcellular localization of BmGalNAcT. (**a**) Schematic representation of the fusion protein of BmGalNAcT_CT_-GFP. The putative cytosolic and transmembrane regions were fused to the N-terminus of GFP. Black dotted, gray, and white boxes indicate the putative cytosolic region, transmembrane region, and catalytic region including the stem region of BmGalNAcT. The numbers indicate the length of each region. (**b**) Dual-color-imaging of BmGalNAcT_CT_-GFP-expressing Sf9 cells stained with organelle dyes. (Top) Imaging of BmGalNAcT_CT_-GFP-expressing cells stained with ER marker. BmGalNAcT_CT_-GFPs without overlapping ER marker are indicated by white triangles (bottom) Imaging of BmGalNAcT_CT_-GFP expressing cells stained with BODIPY TR Ceramide as Golgi marker. Bars: 10 μm.

Next, the subcellular localization of BmGalNAcT was examined using a chimeric protein consisting of the putative cytosolic and transmembrane (CT) regions of BmGalNAcT and GFP (BmGalNAcT_CT_-GFP) (Fig. 1a). BmGalNAcT_CT_-GFP was transiently expressed in Sf9 cells and the cells were co-stained with the ER or Golgi marker. BmGalNAcT_CT_-GFP showed tight co-localization with the Golgi marker (Fig. 1b). On the other hand, some of the GFP signal overlapped a little with the ER marker, some of BmGalNAcT_CT_-GFP did not co-localize with the ER marker and most of the signal was observed around the ER. These results were similar to the localization pattern of *B. mori* α2,6-sialyltransferase^13^. Collectively, they showed that the putative CT regions were sufficient for Golgi localization and that BmGalNAcT was a Golgi-localized membrane protein.

### β1,4-*N*-acetylgalactosaminyltransferase activity of BmGalNAcT

**Figure 2:**
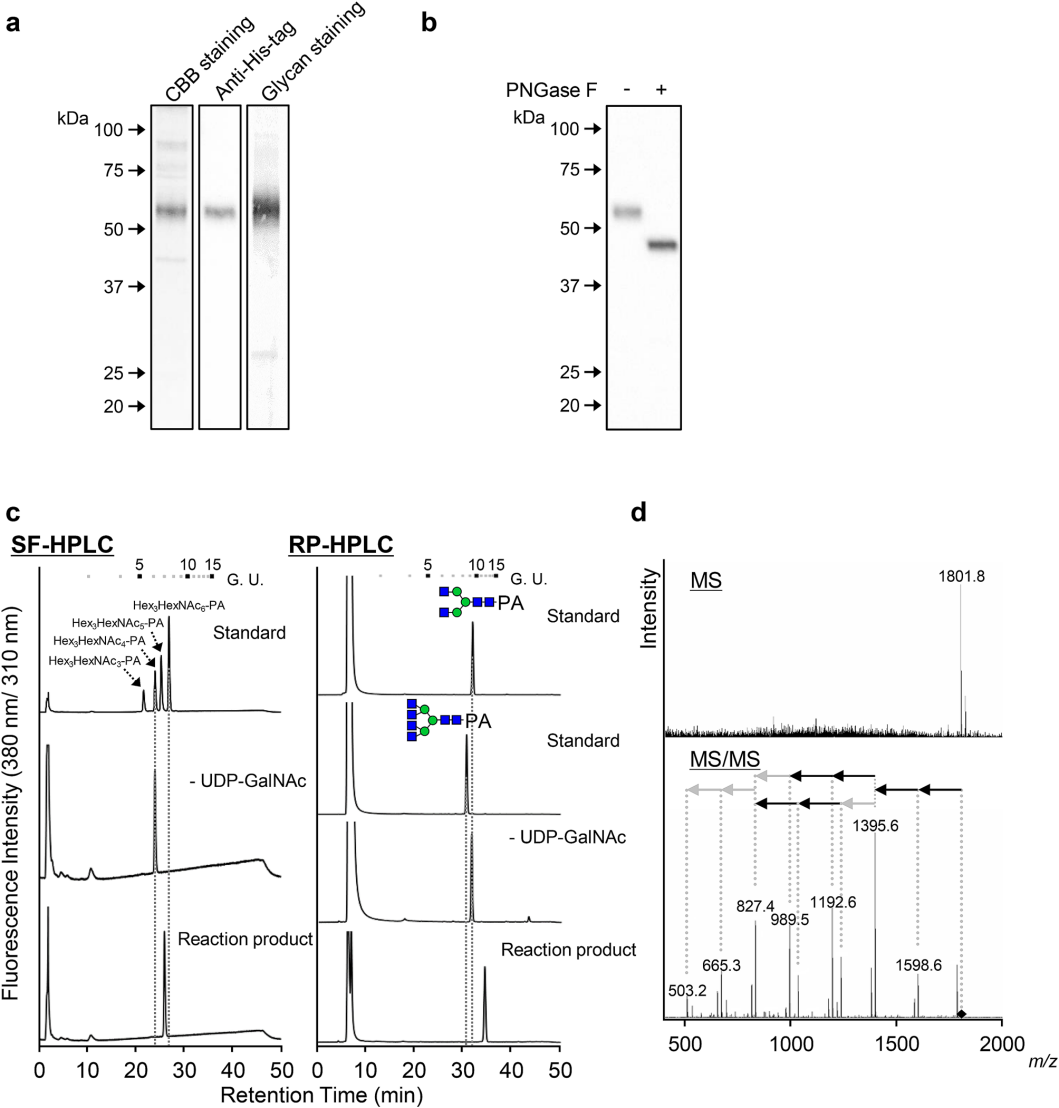
Analysis of the reaction products of BmGalNAcT. (**a**) CBB staining, His-tag staining, *N*-glycoprotein staining, and (**b**) de-glycosylation analysis of purified BmGalNAcT. Black and white triangles indicate the purified *N*-glycosylated form and de-glycosylated BmGalNAcTs, respectively. (**c**) SF- and RP-HPLC analysis of the reaction products. BmGalNAcT reaction was carried out using UDP-GalNAc and GN2M3 as a donor and an acceptor substrate, respectively. The elution position of the product was compared with authentic PA-sugar chains. Numbers at the top represent the elution positions of glucose units on the basis of the elution times of PA-isomalto-oligosaccharides with degrees of polymerization from 3 to 15. Green circles and blue boxes indicate Man and GlcNAc, respectively. (**d**) MS and MS/MS analysis of the reaction product. The MS signal represents (M + H)^+^ ions of the reaction product. The value of *m/z* 503 detected in the MS/MS spectra agrees with the calculated mass of HexNAc_2_-PA. The mass of the precursor ion, *m/z* 1801.8, was considered to correspond to HexNAc_4_Hex_3_HexNAc_2_-PA. The black and gray arrows represent *N*-acetylhexosamine and hexose, respectively. A black diamond indicates the precursor ion of MS/MS fragmentation.

The open reading frame of *BmGalNAcT* was isolated using 5th instar cDNA. The truncated form of *BmGalNAcT* without the CT region was prepared from the full-length cDNA, followed by insertion into a baculovirus expression vector with the N-terminal gp67 sequence and C-terminal His-tag sequence to produce BmGalNAcT as a soluble secreted protein in Sf9 cells. The His-tagged BmGalNAcT was purified from the medium using Co^2+^ affinity chromatography. CBB staining, His-tagged protein detection, and *N*-glycoprotein staining of the purified protein revealed that BmGalNAcT was expressed as an *N*-glycoprotein (Fig. 2a and Supplementary Data 2). Actually, the BmGalNAcT that was de-*N*-glycosylated by PNGase F was approximately 45 kDa (Fig. 2b), which agreed well with the calculated molecular mass of BmGalNAcT without the CT region.

**Figure 3:**
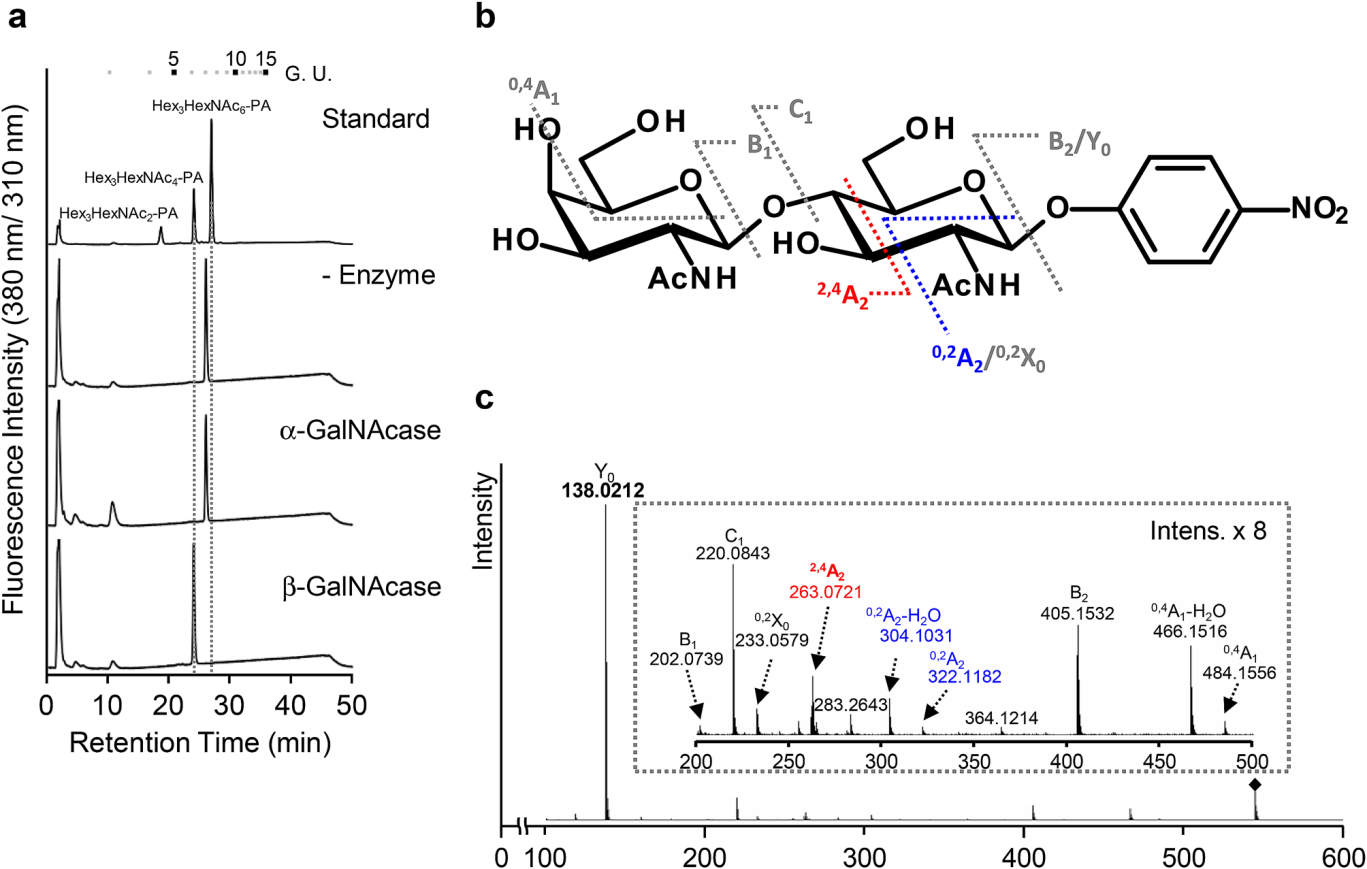
Linkage analysis of the reaction product. (**a**) Linkage-specific *N*-acetylgalactosaminidase digestion of the reaction product. The reaction product purified by SF-HPLC were digested independently by two *N*-acetylgalactosaminidases specific for α- and β- linkage. The digested products were separated by SF-HPLC. Numbers at the top represent the elution positions of glucose units. (**b**) Structural representation and fragmentation scheme of GalNAcβ1,4-GlcNAc-*p*NP. Diagnostic ions are represented in gray characters. (**c**) Negative ion MS/MS fragmentation spectra of *N*-acetylgalactosaminylated GlcNAc-*p*NP. The corresponding *m/z* values of the CID-derived fragment ions and their nomenclatures are assigned in the spectra. The small window shows the enlarged fragment ions in the mass range *m/z* 200–500. *m/z* values shown in black and gray indicate 1,4-linked specific and GalNAcβ-GlcNAc-*p*NP signals, respectively.

The catalytic activity of the purified BmGalNAcT was examined using UDP-GalNAc and the PA-labeled sugar chain GlcNAc_2_Man_3_GlcNAc_2_ (GN2M3-PA) as donor and acceptor substrate, respectively, and the reaction product was analyzed by size-fractionation (SF)-high performance liquid chromatography (HPLC) and reverse phase (RP)-HPLC. The reaction product of BmGalNAcT had a peak that was shifted from the acceptor substrate, which did not correspond to an authentic sugar chain, Hex_3_HexNAc_6_-PA, in either HPLC analysis (Fig. 2c). However, the molecular mass of the reaction product at *m/z* 1801.8 agreed with the calculated mass of Hex_3_HexNAc_6_-PA (Fig. 2d). In addition, the product ions caused by fragmentation in the MS/MS analysis revealed that the reaction product consisted of three Hex and six HexNAc residues (Fig. 2d), demonstrating that BmGalNAcT transferred two GalNAc residues from a donor substrate to the non-reducing terminal of glycan. α- or β-linkage-specific *N*-acetylgalactosaminidase digestion revealed that the GalNAc residues were transferred with β-linkage (Fig. 3a). To ascertain the linkage type by MS/MS analysis in negative mode, the acceptor substrate was changed to *p*-nitrophenyl-GlcNAc (GlcNAcβ-*p*NP). This analysis enabled us to identify the linkage type from the signal pattern of product ions^13^. The molecular mass of the reaction product was *m/z* 544.18, which corresponded to the calculated mass of HexNAc_2_-*p*NP (Fig. 3c). In addition, fragmentation signals from the precursor ion revealed specific signals, i.e., 1,4-linkage *m/z* 263.07 of ^2,4^A_2_ , *m/z* 322.11 of ^0,2^A_2_ and *m/z* 304.1 of ^0,2^A_2_-H_2_O^38,39^ and showed that the fragmentation pattern corresponded to authentic HexNAc1,4-HexNAc-*p*NP rather than HexNAc1,3-HexNAc-*p*NP (Fig. 3b,c). Thus, HexNAc was linked with 1,4-linkage at the non-reducing terminal of GlcNAcβ-*p*NP. These results provided the evidence that BmGalNAcT was the β1,4-*N*-acetylgalactosaminyltransferase contributing to *N*-glycan biosynthesis.

### Acceptor preference of BmGalNAcT

To determine the characteristics of BmGalNAcT, GNM3A (Manα1,6-(GlcNAcβ1,2-Manα1,3)Manβ1,4-GlcNAcβ1,4-GlcNAc-PA; Table 1, Supplementary Fig. S4) was used as a standard acceptor substrate because there was only one terminal GlcNAc residue that was a possible acceptor site, and this assignment facilitated an evaluation of BmGalNAcT activities. The enzymatic properties of BmGalNAcT were as follows: the specific activity was 15.4 nmol/h/mg toward GNM3A, and *K*_m_ and *V*_max_ were 7.8 ± 1.7 µM and 282.8 ± 49.2 nmol/h/mg for GNM3A and 0.77 ± 0.14 mM and 19.4 ± 1.2 nmol/h/mg for UDP-GalNAc (Supplementary Fig. S5). The activities and stabilities under various conditions were analyzed and are summarized in Table 2. Optimal temperature and pH were 20–35 °C and pH 5.5–8.0, respectively. BmGalNAcT showed decreased activities at temperatures higher than 50 °C and pH 7.0 and required Mn^2+^ or Co^2+^ as a cofactor (Supplementary Fig. S6). A similar Mn^2+^ requirement for activity was observed for GT family 7 proteins, especially β1,4-GALT, whereas a Co^2+^ requirement was a unique feature of BmGalNAcT.

**Table 1:** Acceptor substrate specificities of the recombinant BmGalNAcT.

Substrate, structure			Number of terminal GlcNAc	Number of terminal GlcNAc					
				1		2		3	
				Specific activity (nmol/h/mg)	Relative activity (%)	Specific activity (nmol/h/mg)	Relative activity (%)	Specific activity (nmol/h/mg)	Relative activity (%)
*p*NP-sugars									
Monosaccharide		Glcβ-*p*NP		12.3 ± 2.12	0.33				
		Galα-*p*NP		–	–				
		Galβ-*p*NP		–	–				
		GlcNAcβ-*p*NP		3.70 ± 0.05 × 10^3^	100				
		GalNAcβ-*p*NP		–	–				
Disaccharide	Type I	Galβ1,3-GlcNAcβ-*p*NP		–	–				
	Type II (LacNAc)	Galβ1,4-GlcNAcβ-*p*NP		–	–				
	Type III	Galβ1,3-GalNAcβ-*p*NP		–	–				
		GalNAcβ1,3-GlcNAcβ-*p*NP		–	–				
	(LacdiNAc)	GalNAcβ1,4-GlcNAcβ-*p*NP		–	–				
***N*-Glycans**									
Terminal GlcNAc type		GNM3A	1	15.4 ± 0.50	100				
		GNM3B	1	7.92 ± 0.33	51.4				
		GN2M3	2	16.7 ± 0.17	108.2	2.1 ± 0.03	13.6		
		GN3M3	3	21.9 ± 0.20	142.2	10.1 ± 0.40	65.9		
		GN4M3	4	23.3 ± 0.47	151.4	8.3 ± 0.57	53.8	1.0 ± 0.10	6.8
		GN2M3 + bisect GlcNAc	3	–	–				
		GN3M3 + bisect GlcNAc	4	8.27 ± 0.20	53.7				
Core Fucose type		GNM3FA	1	10.2 ± 0.07	66.2				
		GNM3FB	1	9.70 ± 0.27	63.0				
		GN2M3F	2	11.3 ± 0.07	73.5	1.4 ± 0.2	8.8		
		GN2M3F + bisect GlcNAc	3	–	–				
Terminal Gal type		GalGN2M3A	1	3.53 ± 0.20	22.9				
		GalGN2M3B	1	8.29 ± 2.13	53.8				
		Gal2GN2M3	0	–	–				
		GalGN2M3FA	1	–	–				
		GalGN2M3FB	1	8.02 ± 1.47	52.1				
Mannose type		M3	0	–	–				
		M5	0	–	–				

Relative rates were calculated on the basis of the activity toward GNM3A (100%).

**Table 2:** Enzyme properties of recombinant BmGalNAcT expressed in Sf9 cells.

	Optimal condition
Optimum pH	5.5–8.0
pH stability	4.0–7.0
Optimum temperature	20–35 °C
Temperature stability	0–50 °C
**Metal-ion dependence : relative activity in the presence of**	
No addition	2.3 ± 1.4%
10 mM MgCl_2_	3.9 ± 1.4%
10 mM MnCl_2_	100%
10 mM CaCl_2_	12.4 ± 0.7%
10 mM CoCl_2_	91 ± 0.1%
10 mM ZnCl_2_	1.2 ± 0.2%

The relative activities in metal-ion dependency were calculated on the basis of the presence of Mn^2+^ (100%).

The acceptor substrate specificities were examined using various kinds of synthetic *p*NP substrates or PA-labeled sugar chain candidates (Table 1, Supplementary Fig. S4). BmGalNAcT transferred GalNAc to Glcβ-*p*NP and GlcNAcβ-*p*NP, but the transfer efficiency was much higher efficiency toward GlcNAcβ-*p*NP than toward Glcβ-*p*NP. In case of *N*-glycan substrate, BmGalNAcT preferred GlcNAc residue(s) at the non-reducing terminal of the core Manα1,3-Man-R residue, but all GlcNAc residues were utilized as target residues for GalNAc transfer(s). In comparison with GNM3A and GalGNM3B or GNM3B and GalGNM3A, the relative activities were approximately half due to the presence of the β1,4-linked Gal residue. It is noteworthy that the transfer activity was decreased by the α1,6-fucose (Fuc) residue and was strictly inhibited by the bisected GcNAc residue even though the α1,6-Fuc residue, in particular, was not proximal to the non-reducing terminal of GlcNAc residue(s). Three-dimensional *N*-glycan modeling revealed that α1,6-Fuc was apart from the β1,2-linked GlcNAc residue(s) and that the bisected GlcNAc residue was clearly localized in close proximity to the target GlcNAc residues and faced the same side of target β1,2-linked GlcNAc residues (Supplementary Fig. S7). These findings suggested that α1,6-Fuc, when close to the active site, interfered with the access of the acceptor *N*-glycan to BmGalNAcT, whereas the bisected GlcNAc residue prevented the access of donor substrates to BmGalNAcT. On the other hand, GN3M3 with bisected GlcNAc was utilized as an acceptor. Moreover, the specific activity toward GN3M3 was higher than that toward GN2M3. These results suggested that the GalNAc was preferentially transferred to the β1,4-linked GlcNAc residue at the non-reducing terminal of the core Manα1,3-Man residue. This substrate preference was also observed for GN4M3. This result was supported by the two results. First, GN2M3 with bisected GlcNAc was not available as an acceptor substrate, but GN3M3 with bisected GlcNAc did function as an acceptor substrate by the additional β1,4-linked GlcNAc on the core Manα1,3-Man residue of GN2M3. Second, only the β1,4-linked GlcNAc residue was located outside of the face of GN2M3 with bisected GlcNAc (Supplementary Fig. S7b,c). These results indicated that the β1,4-linked GlcNAc residue was permitted to access the donor substrate in an active site and was recognized as a target residue of GalNAc transfer. Therefore, GalNAc was transferred selectively to the β1,4-linked GlcNAc residue.

### Gal and GlcNAc transfer activity of BmGalNAcT

**Figure 4:**
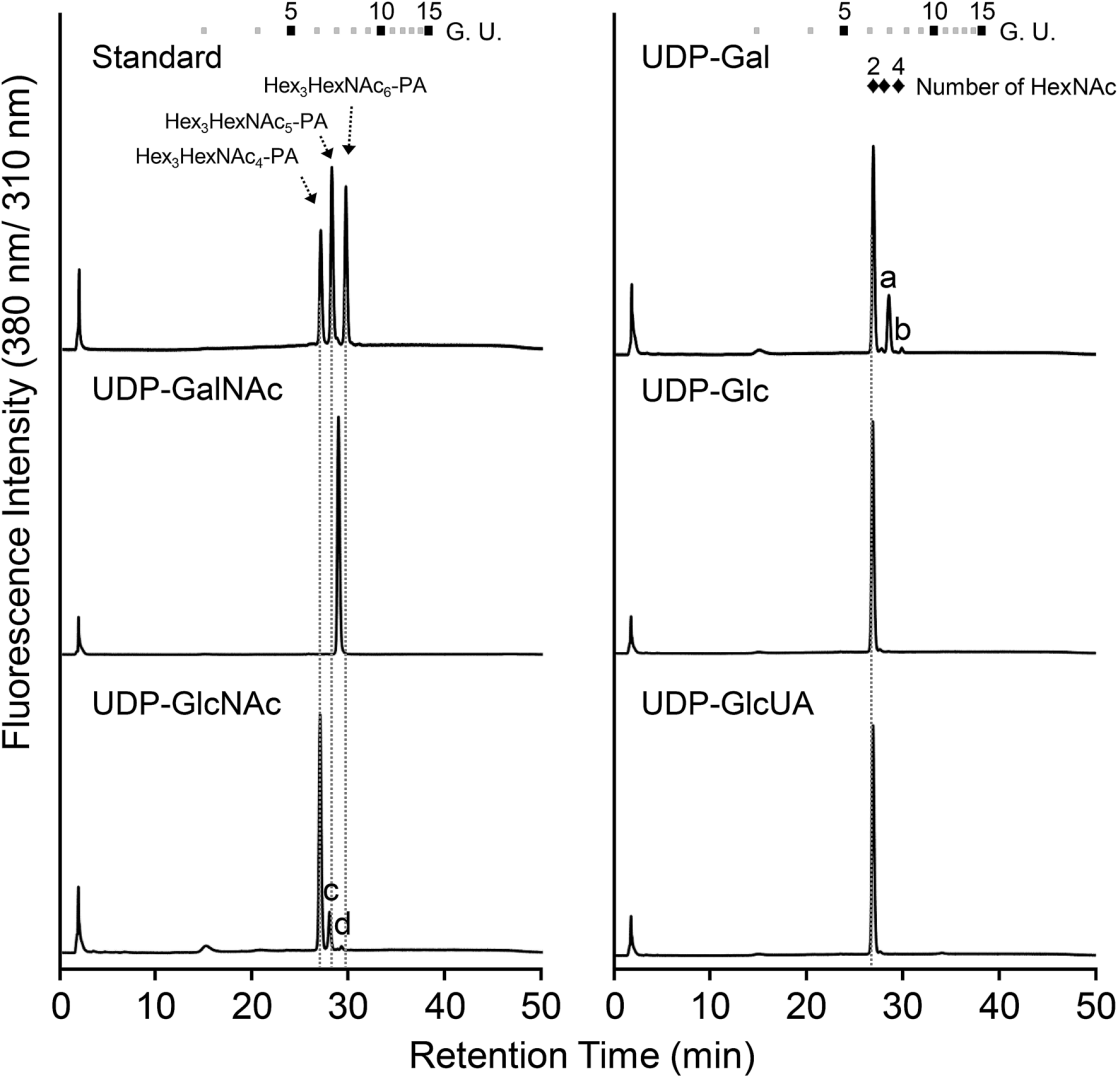
RP-HPLC analysis of the products of BmGalNAcT reaction using various donor substrates. Reaction products were separated by SF-HPLC. Peaks a and b in UDP-Gal and peaks c and d in UDP-GlcNAc were collected and used for further analysis. Boxes and diamonds with numbers at the top represent the elution positions of glucose units and *N*-acetylglucosaminylated PA-sugar chains, respectively.

A previous report revealed that TnGalNAcT transferred Gal and GlcNAc to a synthetic acceptor substrate but had little or no activity toward *N*-glycan^24^. To ascertain whether this property of TnGalNAcT applied to BmGalNAcT, BmGalNAcT reaction was performed in the presence of GN2M3 or GlcNAcβ-*p*NP as acceptor substrates and a series of UDP-sugar nucleotides, i.e., UDP-Gal, UDP-GlcNAc, UDP-glucose (Glc), or UDP- glucuronic acid (GlcUA) as donor substrates. Surprisingly, BmGalNAcT mediated sugar transfer from UDP-Gal or UDP-GlcNAc and synthesized chain-length elongated *N*-glycan or GlcNAcβ-*p*NP, thereby resulting in antennal extension of *N*-glycans and formation of chitobiosyl-*p*NP (Fig. 4, Supplementary Fig. S8), however, the specific activities of BmGalNAcT using UDP-Gal and UDP-GlcNAc as a donor substrate were quite different from that of UDP-GalNAc. Specific activities using UDP-GalNAc, UDP-Gal, and UDP-GlcNAc as donor substrates were 3.70 ± 0.05 × 10^3^ nmol/h/mg, 158 ± 3.2 nmol/h/mg, 65.8 ± 1.89 nmol/h/mg, respectively. Focusing on the reaction product using UDP-Gal as a donor substrate, MS and MS/MS analysis and SF-HPLC analysis of the reaction product using GN2M3 as an acceptor substrate exhibited that the molecular mass and the retention of the predominant product (peak a in Fig. 4) corresponded to Galβ1,4-GN2M3 (Supplementary Fig. S9a,b). Linkage-specific galactosidase digestion also revealed that Gal residues were transferred with β1,4-linkage (Supplementary Fig. S9c). In addition, the *N*-acetylhexosaminidase (HEXO)-digested peak a corresponded to authentic PA-sugar chains, GalGNM3A and GalGNM3B, at approximately the same ratio (Supplementary Fig. S9d), indicating that BmGalNAcT had the potential to transfer a Gal residue to both non-reducing termini of bi-antenna GlcNAc on sugar chain. Indeed, BmGalNAcT synthesized Gal2GN2M3 (peak b in Fig. 4, Supplementary Fig. S10).

**Figure 5:**
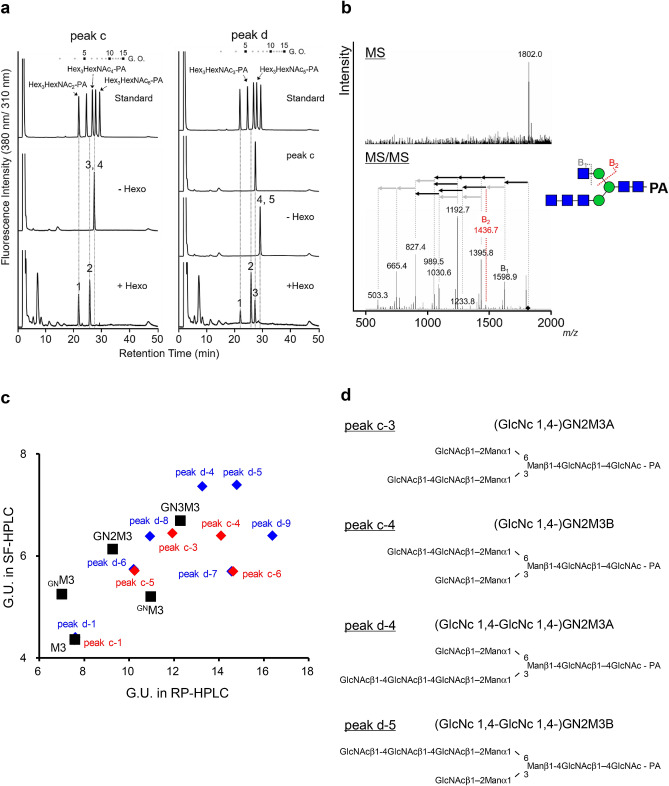
Structural determination of *N*-acetylglucosaminylated sugar chains. (**a**) SF-HPLC analysis of *N*-acetylhexosaminidase-digested peaks c and d. (**b**) MS and MS/MS analysis of peak d-4. The putative structure of peak d-4, fragmentation scheme, and diagnostic ions are represented. (**c**) Two-dimensional mapping of peak c, hexosaminidase-digested peak c, peak d, hexosaminidase-digested peak d, and authentic PA-sugar chains. Elution positions were calculated from the glucose unit in RP-HPLC and SF-HPLC. The red diamond, blue diamond, and black box indicate peak c, peak d in Figure, their *N*-acetylhexosaminidase (HEXO)-digested products, and the authentic PA-sugar chain, respectively. (**d**) Deduced structures of *N*-acetylglucosaminylated GN2M3.

In the GlcNAc transfer, the reaction product showed that BmGalNAcT also mediated GlcNAc transfer to GN2M3 and GlcNAcβ-*p*NP (peak c and d in Fig. 4, Supplemental Fig. S8). However, the retention time of the products and the HEXO-digested products in SF-HPLC were different from those of authentic bi-, tri-, and tetra-antennary GlcNAc carrying PA-sugar chains (Fig. 5a), suggesting that BmGalNAcT transferred GlcNAc residue(s) not to Man residue(s), but possibly to terminal GlcNAc residue(s). MS, MS/MS analysis, and RP-HPLC analysis of the predominant product peak c demonstrated that the reaction product carried GlcNAc residue(s) at two different positions on GN2M3 (Supplementary Fig. S11a,b). The reaction products did not correspond to either bisected GN2M3 or GN3M3 as shown in Supplementary Fig. S11b. This suggested that BmGalNAcT transferred two GlcNAc residues to the non-reducing terminal of GlcNAcβ1,2-Man-R on *N*-glycan. An unknown linkage of the GlcNAc residue was HEXO-sensitive, but the structure of the reaction product did not correspond to GN2M3 in SF-HPLC (Fig. 5a). The HEXO-digested reaction product also showed two peaks in RP-HPLC, which had different hydrophobicities from GN2M3 (Supplementary Fig. S11c), indicating that the GlcNAc residue linked at two different positions and that HEXO preferentially digested the β1,2-GlcNAc residue on GN2M3. These results led to speculation that the putative HEXO-digested structures were (GlcNAcβ1,4-)GNM3A and (GlcNAcβ1,4-)GNM3B (Fig. 5d). Thus, the GlcNAc-transferred structures of peak c were (GlcNAcβ1,4-)GN2M3A and (GlcNAcβ1,4-)GN2M3B. The ratio of (GlcNAcβ1,4-)GN2M3A and (GlcNAcβ1,4-)GN2M3B synthesized by BmGalNAcT was approximately the same: a molar ratio of 54.8%:45.2%. Importantly, BmGalNAcT transferred two GlcNAc residues to different positions on GN2M3 (peak d in Fig. 4, Supplementary Fig. S11d). This was quite different from the results for the reaction of BmGalNAcT using UDP-GalNAc as a donor substrate, which yielded GalNAc2GN2M3 as the only reaction product (Fig. 2c). To determine these structures, additional RP-HPLC analysis and MS/MS analysis were performed (Fig. 5b and Supplementary Fig. S11d,e). The peak d had two peaks in RP-HPLC analysis, indicating that two GlcNAc residues were transferred to a GN2M3 position different form that for tetra-antenna GN4M3. Furthermore, the second product ion from the precursor, B_2_ *m/z* 1436.7, on MS/MS analysis demonstrated that core mannose residues were masked with only one GlcNAc residue and that at least one terminal GlcNAc residue on GN2M3 was modified with serially concatenated GlcNAc residues. Interestingly, 2D-mapping of PA-sugar chains based on retention times in SF-HPLC and RP-HPLC demonstrated that the HEXO product of peak d did not correspond to any PA-sugar chains of the BmGalNAcT reaction product or the HEXO products (Fig. 5c). Thus, two GlcNAc residues were not transferred to either β1,2-linked GlcNAc residue but rather were utilized to synthesize (GlcNAcβ1,4-GlcNAcβ1,4-)GN2M3A or (GlcNAcβ1,4-GlcNAcβ1,4-)GN2M3B (Fig. 5d). Unfortunately, the amount was too small, the further structural analysis was unable to be carried out, however, it is predicted that the structures contained a novel linkage type of GlcNAc. These results demonstrated that although the specific activities of Gal and GlcNAc transfer were not determined due to the quite low activities, BmGalNAcT clearly functioned as β1,4-GALT and β1,4-*N*-acetylglucosaminyltransferase.

### *N*-Glycan analysis of BmGalNAcT-expressing insect cells

**Figure 6:**
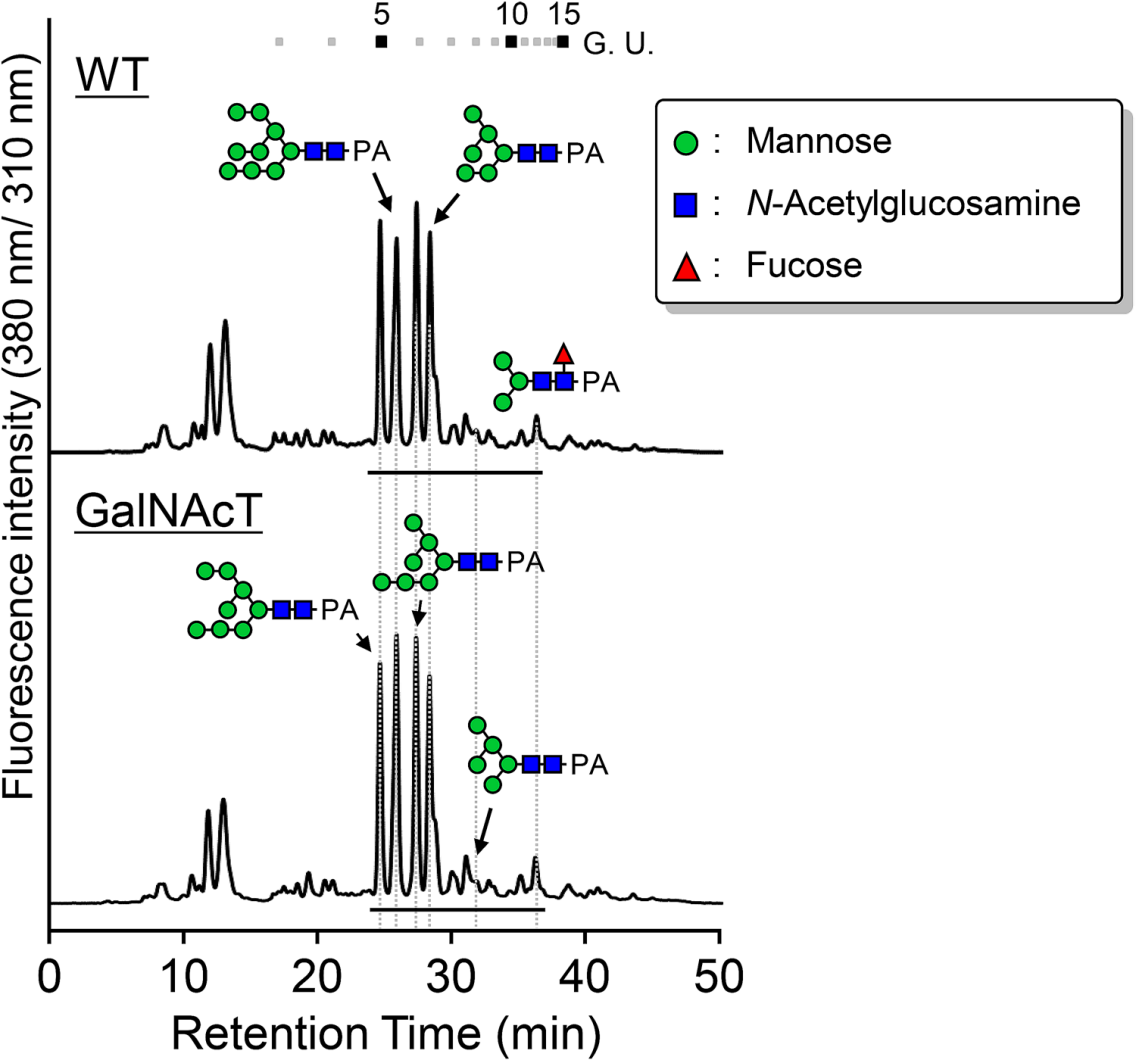
*N*-Glycan analysis of WT and BmGalNAcT-expressing Sf9 cells. Total soluble sugar chains prepared from glycoproteins and labeled with PA were analyzed by RP-HPLC. The major structures are shown in chromatographs. Underlining indicates the PA-sugar chain fraction applied to LC–MS/MS analysis.

Previous studies had identified β1,4-*N*-acetylgalactosaminylated *N*-glycans in insect cells^15–21^. Interestingly, expression of *T. ni* GalNAcT in Sf9 cells resulted in the production of terminal β1,4-*N*-acetylgalactosaminylated *N*-glycans in vivo^24^. We therefore reasoned that, if BmGalNAcT has a functional in vivo activity in Sf9 cells, the structure of *N*-glycan would be terminally modified with GalNAc residue(s). To investigate this hypothesis, full length *BmGalNAcT* was introduced into Sf9 cells. BmGalNAcT was successfully expressed in Sf9 cells as an *N*-glycoprotein and the cells possessed the same level of GalNAcT activity as seen in secreted forms of BmGalNAcT (Supplementary Fig. S12 and Supplementary Data 3). To determine the major *N*-glycan structures in greater detail, the neutral *N*-glycan structures attached at total soluble *N*-glycoproteins were determined by hydrazinolysis of *N*-glycoprotein, followed by PA-labeling, RP-HPLC, and LC–MS/MS analysis (Fig. 6, and Supplementary Table S1). There was no significant *N*-glycan structural difference between mock cells and BmGalNAcT-expressing cells: the predominant structures were of high mannose type, whereas complex-type structures were hardly detected. Moreover, BmGalNAcT-expressing cells did not synthesize detectable levels of endogenous terminal β1,4-*N*-acetylgalactosaminylated *N*-glycoprotein. These results indicated that BmGalNAcT has β1,4-*N*-acetylgalactosamine activity in vitro but the overexpression of BmGalNAcT itself did not result in a *N*-glycosylation change in insect cells.

## Discussion

In general, complex-type *N*-glycan exists in a state in which sialic acid is transferred to galactose. Sialylated *N*-glycan in mammals is synthesized by sequential reactions mediated by galactosyltransferase and sialyltransferase. Neither gene presents alone, such that the presence of either gene indicates the possibility of the presence of the other gene. Previously, the presence and the activity of *B. mori* ST were identified, suggesting that *B. mori* has the transferase classified as the GT7 family protein and transfers the Gal and/or GalNAc residue to *N*-glycan. In addition, Dojima et al. showed that *B. mori* larvae had β1,4-galactosylated *N*-glycan, even though *B. mori* β1,4-GALT has not been identified^40^. Thus, the enzyme contributing to β1,4-galactosylation in *B. mori* larvae was speculated to be GalNAcT, instead of β1,4-GALT, from the evolution of the enzyme and the presence of another insect GalNAcT. Besides, to date, the LacdiNAc structure has also been found not only on human glycoproteins^41^, but in insects i.e., in *Drosophila*, mosquito, honeybees, and lepidopteran^15–21^ and in glycolipids^42^. Recently, *B. mori* GalNAcT was identified^34^, and therefore, BmGalNAcT might synthesize uncharacterized and unique *N*-glycan structure(s). These pieces of evidence encouraged us to further characterize *B. mori* GalNAcT as also the enzyme was already identified.

Here, focusing on the potential for synthesizing potential complex *N*-glycan(s) in silkworms, we identified and characterized *B. mori* Golgi-localized *N*-acetylgalactosaminyltransferase. The donor substrate determinant of GT7 in BmGalNAcT was Ile310, which resulted in the transfer of GalNAc preferentially to the sugar residue on the sugar chain by β1,4-linkage rather than Gal. To reveal the presence of all BmGalNAcT homologues, we reinvestigated *B. mori* genome. Putative β1,4-*N*-acetylgalactosaminyltransferase (KWMTBOMO10034) encoding *bre-4* has higher homology to BmGalNAcT than any other candidates. *B. mori* bre-4 has some of the highly conserved sequences in GT family 7 proteins, such as the site binding both the donor and acceptor substrates, and the ion binding site. However, the other sequences except for the conserved sequences did not corresponded to other insect GalNAcTs. In addition, XP_004926306, assigned as β1,4-galactosyltransferase 1-like on the database, is another candidate for *B. mori* GalNAcT, but it also lacked most of the conserved sequences of GalNAcT. These results indicated that *B. mori* possesses a single GalNAcT gene with a potential role in *N*-glycosylation.

BmGalNAcT activity was suppressed by the presence of α1,6-Fuc and/or β1,4-Gal residue(s) and strictly inhibited by a bisected GlcNAc residue. Even though weaker efficiency than GalNAc transfer, BmGalNAcT could also produce the β1,4-galactosylated sugar chain, which is distinct from the properties of TnGalNAcT, which shows no ability to transfer Gal to *N*-glycan^24^. In addition to GalNAc and Gal, BmGalNAcT transferred GlcNAc to the sugar chain and *p*NP-sugar, but not Glc and glucuronic acid GlcUA. This broad substrate specificity was not observed in the previous report^34^. Since this GlcNAc transfer to *N*-glycan has not been reported, it could be a unique feature of BmGalNAcT. These results suggest that the donor substrate is required; BmGalNAcT recognizes both the axial OH group at the C4-position and/or acetoamide group at the C2-position on the sugar residue of the donor substrate for transferring the sugar residue to the acceptor substrate.

TnGalNAcT had in vivo activity and produced *N*-acetylgalactosaminylated and β1,4-galactosylated *N*-glycoprotein, suggesting that insect cells had the potential to biosynthesize *N*-acetylgalactosaminylated and β1,4-galactosylated *N*-glycan even though TnGalNAcT had no in vitro activity toward *N*-glycan^24^. In fact, at least one donor substrate, UDP-Gal, in insect cells is abundant^43^. Therefore, overexpression of GT7 proteins in insect cells results in successful biosynthesis of β1,4-galactosylated *N*-glycan without the addition of UDP-Gal^44–46^. However, our detailed total *N*-glycan analysis was unable to detect either *N*-acetylgalactosaminylated or β1,4-galactosylated *N*-glycan in major neutral glycan fraction. This may have been because the weak β1,4-GALT activity of BmGalNAcT was insufficient to produce β1,4-galactosylated *N*-glycan in comparison with GalNAcT activity. In addition, both mock and BmGalNAcT-expressing Sf9 cells had high-mannose type structures as a predominant *N*-glycan. It is possible that high-mannose *N*-glycans were immature in *N*-glycosylation. Indeed, though mock cells had high-mannose type *N*-glycan only, stable expression of a mammalian glycosyltransferase, *N*-acetylglucosaminyltransferase II (GNTII), in Sf9 cells resulted in the production of complex-type *N*-glycan^47^. Therefore, even for purposes of elucidating the effects of *N*-acetylgalactosaminylation of *N*-glycan on physiological and biological functions in insects, introduction of both BmGalNAcT and GNTII would be necessary for the efficient production of *N*-acetylgalactosaminylated *N*-glycan. Interestingly, previous studies represented that insect has *N*-acetylgalactosaminylated *N*-glycans^16–21^. In this study, we investigated the major *N*-glycan structures and could not detect *N*-acetylgalactosaminylated *N*-glycans. However, BmGalNAcT-expressing Sf9 cells might have anionic *N*-glycans with glucuronic acid, sulphation, phosphorylcholine, and/or phosphoethanolamine as observed in other insects^18–21^ and *N*-acetylgalactosaminylated *O*-glycans and glycolipid because of the possibility that some GT7 proteins contribute to *O*-glycans and glycosphingolipid biosynthesis^48^.

It is worth noting that BmGalNAcT transferred GlcNAc residues in a different manner from GalNAc residues; BmGalNAcT synthesized structures of (GlcNAcβ1,4-GlcNAcβ1,4-)GN2M3 (Fig. 5d) instead of bi-antenna (GlcNAcβ1,4-GlcNAc)2M3. This suggests that BmGalNAcT prefers β1,4-GlcNAc residue to the β1,2-GlcNAc residue at the non-reducing terminus as an acceptor in the case of GlcNAc transfer. However, we observed that the reaction product transferred only two GlcNAc residues (Fig. 4). This was considered to be due to the weak GlcNAc transfer activity. On the other hand, GalNAcβ1,4-GlcNAc was detectable, whereas GalNAcβ1,4-GalNAc was not detected. These results provided evidence that BmGalNAcT recognizes the GlcNAc residue as an acceptor sugar residue. This idea was further supported by the result that BmGalNAcT transfers a GalNAc residue to the β1,4-linked GlcNAc residue on GN3M3 (Table 1). However, such recognition would not apply to bisected GlcNAc due to *N*-glycan conformation (Supplementary Fig. S7). Here, one question arises. Can BmGalNAcT could the repeating unit(s) of GalNAcβ1,4-GlcNAc, (GalNAcβ1,4-GlcNAc)_n_, on GN2M3? It is assumed that various conditions must be met to biosynthesize the repeating unit, but introduction of *Caenorhabditis elegans* GalNAcT into mammalian CHO cells resulted in the successful production of α1,3-fucosylated (GalNAcβ1,4-GlcNAc)_n_M3^49^. Thus, BmGalNAcT also possesses the potential to synthesize the repeating unit(s) of (GalNAcβ1,4-GlcNAc)_n_ on *N*-glycan. To achieve this and analyze the in vivo function of not only *N*-glycan *N*-acetylgalactosaminylation but also poly GalNAcβ1,4-GlcNAc on *N*-glycan, the creation of enzymes with higher activity of GlcNAc transfer, i.e., protein engineering, and establishment of a supplemental pathway for a donor substrate of UDP-GalNAc are required. There are no reports focusing on the production of UDP-GalNAc in *B. mori*, but as a first effort in this direction, the presence of UDP-GalNAc and the genes involved in biosynthesis of UDP-GalNAc should first be clarified.

Complete loss of UDP-galactose 4′ epimerase of Drosophila is embryotic lethal in fruit flies^50^. On the other hand, GalNAcT deficiency in insects leads to the display of differentiation and developmental phenotypes, but not lethality^31,51^, suggesting that UDP-GalNAc is essential for biosynthesis of glycolipids and *O*-glycans rather than *N*-glycans. What is the in vivo function of BmGalNAcT? To investigate this question, there is need of an effective knockout strategy. Genome editing technologies, such as the ZFN, TALEN and CRISPR/Cas9 platforms, have been established for silkworm, and have led to a remarkable acceleration of *B. mori* research^52–55^. However, these technologies have not been applied to knockout of the glycosyltransferases responsible for *N*-glycan biosynthesis and maturation, including BmGalNAcT and BmST. Furthermore, overexpression of these proteins in insects has not been performed. Such overexpressions or knockouts of glycogenes of unknown function might lead to the uncovering of a positive and/or negative effect on *B. mori* growth, morphological features and physiological changes. Our recent study demonstrated that only MSG shows drastic change though the stage of fifth larvae, but accumulates a high level of *N*-acetylglucosaminylated *N*-glycan at the stage of fifth larvae, which accounted for more than 50% of total *N*-glycan in some cases, whereas other organs, such as the gut, posterior silk gland, fat body, and body fluid, accumulate high-mannose type structures as predominant structures and have a small amount of *N*-acetylglucosaminylated *N*-glycan (data not shown). Thus, in the same manner as the nervous system, MSG in *B. mori* fifth larvae seems to be the organ rendered most susceptible by overexpression and knockout of BmGalNAcT and BmST. The UAS/GAL4 system, which was widely used for organ-specific gene expression, has also been considered to be useful for both genetic approaches to specifically control gene expression in MSG. There is still room for elucidation of *N*-glycosylation in *B. mori*, and the *N*-glycosylation function(s) related to insect differentiation and growth should be further elucidated.

## Methods

### Materials

UDP-GalNAc, UDP-Gal, UDP-GlcNAc, UDP-Gal, and UDP-GlcUA were from YAMASA (Chiba, Japan). *p*-Nitrophenyl (*p*NP)-GlcNAc was purchased from Tokyo Chemical Industry (Tokyo, Japan). 2-Pyridylaminated (PA)-sugar chains were purchased from TaKaRa Bio (Shiga, Japan) and Masuda Chemical Industry (Kagawa, Japan). *Streptococcus pneumoniae* β-*N*-Acetylgalactosaminidase was purchased from Sigma (St. Louis, MO). ER-Tracker Red and BODIPY TR Ceramide Complex to BSA was purchased from Molecular Probes (Eugene, OR).

### Insect cells

*Spodoptera frugiperda* Sf9 cells were maintained at 25 °C in Sf-900 III SFM (Gibco, Eggenstein, Germany) containing 10% fetal calf serum (PAA Laboratories GmbH, Pashing, Austria).

### Identification, isolation and cloning of *BmGalNAcT*

BmGalNAcT was identified using KAIKOBase (http://sgp.dna.affrc.go.jp/KAIKO/jp/index.ht ml) and human β1,4-Galactosyltransferase I (NP_001488), Drosophila (AAD34746 and AAF56843), and *Trichoplusia ni* (AAT11926) GalNAcTs as queries.

*Bombyx mori* fifth instar larvae total RNA was isolated using an RNeasy Plant Mini Kit (QIAGEN, Chatsworth, CA), followed by reverse transcription using a PrimeScript RT reagent Kit (TaKaRa). The full-length *BmGalNAcT* cDNA was amplified using KOD plus polymerase (ToYoBo, Osaka, Japan), cDNA, and the following primer set: *BmGalNAcT*-Fw, 5′-GCTTGGTCATCGCATGCG-3′; *BmGalNAcT*-Re, 5′-ATACCTCGCCAAGCTGCTGT-3′. The PCR product was subcloned into pGEM T-Easy vector (Promega, Madison, WI), followed by sequencing using an ABI PRISM Big Dye Terminator cycle sequencing kit (Applied Biosystems, Foster City, CA).

### RNA extraction and quantification of *BmGalNAcT* expression

Total RNA was isolated from the middle silkgland, posterior silkgland, fat body, body liquid, middle gut, Malpighian tubule, and testis in 5th instar larvae, and cDNAs were synthesized as described above. The expressions of *BmGalNAcT* were estimated by amplification using the following primer sets: forward, 5′-AGAGATCGCCAACAGCACTTG-3′; reverse, 5′-TCCAGTCTCTGGCTCTCGAT-3′. As a control, *Bmrp49* was amplified using following primer set: forward, 5′-CAGGCGGTTCAAGGGTCAATAC-3′; reverse, 5′-TGCTGGGCTCTTTCCACGA-3′.

### Subcellular localization analysis

A chimeric construct of BmGalNAcT_CT_-GFP was generated by PCR using full-length GmGalNAcT and GFP as templates. The fusion construct was ligated into pFastBac vector (Invitrogen, Carlsbad, CA). Baculovirus for the production of BmGalNAcT_CT_-GFP was prepared using the Bac-to-Bac expression system and FuGENE Transfection Reagent (Promega). Sf9 cells were transfected using P2 baculovirus and grown at 25 °C for 3 days. The BmGalNAcT_CT_-GFP cells were fixed with 4% paraformaldehyde in phosphate-buffered saline (PBS) for 20 min, followed by washing with PBS three times, and staining using organelle dye ER-Tracker Red or BODIPY TR Ceramide in PBS for 15 min. The cells were washed with PBS three times and then the fluorescence signals were observed under a Leica DMI4000B equipped with TCS-SPE (Leica Microsystems, Heidelberg, Germany), and LAS AF software (Leica Microsystems). Fluorescence was excited with the 488-nm and the 532-nm lines of solid lasers. Image processing for GFP and organelle dye coloration was performed using Adobe Photoshop CS4.

### Heterologous expression and purification of BmGalNAcT

Full-length *BmGalNAcT* with or without a putative cytoplasmic and transmembrane region was amplified using KOD plus polymerase, full-length cDNA as a template and the following primer set (forward: 5′-ATGGATCCATGGGAGCGGCGCG-3′ for full-length BmGalNAcT expression and ATTGAATTCGACGCCTCGCCGCTC for expression of the truncated form of BmGalNAcT; reverse: 5′-ATAAGCTTCTAGTGATGGTGATGGTGATGGCTGCGCTCATCGATG-3′). The truncated form of *BmGalNAcT* with a GP67 signal at the 5′-upstream region was introduced into the pFastBac vector (Invitrogen) to produce BmGalNAcT as a soluble and secreted protein.

Baculovirus for the production of BmGalNAcT was prepared as described above. After amplification of the P2 baculovirus, BmGalNAcT was expressed in Sf9 cells as previously reported^13^. In brief, a total of 1.0 × 10^8^ Sf9 cells in 100 ml medium were transfected with P2 baculovirus and cultivated at 25 °C, 120 rpm, for 4 days. The medium was collected and centrifuged at 4 °C, 1000*g* for 5 min. To precipitate proteins, ammonium sulfate precipitation was performed. The precipitant was dissolved in 50 mM Tris–HCl buffer, pH 7.5, 500 mM NaCl, and 5 mM imidazole (buffer A), followed by dialysis against buffer A overnight. The solution was subject to a TALON Resin column (TaKaRa) equilibrated with buffer A. After washing with a 50-times-column volume of buffer A, the recombinant enzyme was eluted with buffer A containing 200 mM imidazole. The eluate was dialyzed against 40 mM Tris–HCl pH 7.5, 300 mM NaCl (buffer B) for 3 h, followed by measurement of the protein concentration and an addition of glycerol and buffer B to prepare 0.15 mg/ml of BmGalNAcT at a final concentration of 20 mM Tris–HCl pH 7.5, 150 mM NaCl, 50% glycerol. The enzyme solution was stored at – 20 °C.

### *N*-Glycosylation analysis of BmGalNAcT

The purified BmGalNAcT was separated by 5–20% SDS-PAGE and detected by CBB staining, glycan detection using G.P.Sensor (J-OIL MILLS, Tokyo, Japan), or western blotting. In western blotting analysis, anti-HRP antibody and anti-rabbit antibody conjugated to horseradish peroxidase (GE Healthcare, Tokyo, Japan) were used as a primary antibody and a secondary antibody, respectively, and specific signals were visualized using Luminate Forte western HRP Substrate (MILLIPORE, Billerica, MA) and a ChemiDoc MP system (Bio-Rad, Hercules, CA).

The purified BmGalNAcT were digested with Peptide:*N*-glycosidase F (PNGase F, TaKaRa) or following the manufacturer's protocols, followed by separation by 5–20% SDS-PAGE and visualization by western blotting.

### BmGalNAcT assay

Basic BmGalNAcT assays were performed in 20 µl of total reaction volume containing 10 mM cocodylic acid buffer, pH 7.5, 10 mM MnCl_2_, 5 mM UDP-GalNAc, 10 pmol 2-aminoprydine (PA)-labeled GNM3A, GN2M3 or 5 mM GlcNAcβ-*p*NP and 0.30 µg of purified BmGalNAcT at 25 °C for 2 h. The reaction was terminated by incubation at 100 °C for 5 min. The samples were centrifuged at 4 °C, 20,000*g* for 5 min. The supernatant was subject to HPLC analysis.

### HPLC analysis

The reaction products of the PA-sugar chains and GlcNAcβ-*p*NP were detected by an SF-HPLC and/or RP-HPLC using a HITACHI LaChrom HPLC System equipped with a fluorescence or a UV detector, respectively. The PA-sugar chains of reaction products were separated using the mobile phase of acetonitrile/acetic acid (solvent A: 98/2, v/v) and water/acetic acid/triethylamine (solvent B: 92/5/3, v/v/v). The PA-sugar chain was separated using a Shodex Asahipak NH2P-50 2D column (2.0 mm ID × 150 mm; SHOWA DENKO Co., Ltd.) by linearly increasing the solvent B concentration from 20 to 55% over 35 min at a flow rate of 0.2 ml/min. The eluted fractions were monitored by measuring the fluorescence intensity using excitation and emission wavelengths of 310 and 380 nm, respectively. The reaction products of *p*NP derivatives were separated using 0.02% trifluoroacetic acid (TFA) (solvent C) and methanol/0.02% TFA (solvent D: 10/90, v/v) using a Mightysil RP-18 GP column (4.6 mm × 250 mm, Kanto Chemical Co., Tokyo, Japan) with an HITACHI LaChrom HPLC System by linearly increasing the solvent D concentration from 0 to 15% over 7 min. The eluted fractions were monitored by measuring the UV intensity at a wavelength of 300 nm. The reaction product of GlcNAcβ-*p*NP for MS/MS analysis was purified using the mobile phase of 50 mM CH_3_COONH_4_/acetonitrile (87/13, v/v) by Cosmosil 5C18-AR-II column (6.0 × 250 mm; Nacalai Tesque, Kyoto, Japan) at an isocratic flow rate of 1.2 ml/min.

The structural determination was performed using RP-HPLC. The mobile phase was composed of 0.02% TFA (solvent E) and acetonitrile/0.02% TFA (solvent F) (20/80, v/v). RP-HPLC was performed using a Cosmosil 5C_18_-AR-II column (4.6 × 250 mm; Nacalai Tesque, Kyoto, Japan) with an HITACHI LaChrom HPLC System by linearly increasing the solvent F concentration from 0 to 20% over 35 min at a flow rate of 0.7 ml/min. The eluted fractions were monitored by measuring the fluorescence intensity using excitation and emission wavelengths of 310 and 380 nm, respectively.

### Linkage analysis by mass spectrometry and exoglycosidase digestion

In the RP-HPLC analysis, the reaction product, GalNAc-GlcNAcβ-*p*NP, was collected and lyophilized. The resultant product was dissolved in 50% acetonitrile prior to the mass spectrometry (MS) analysis. The molecular mass of the reaction product was determined by direct infusion into a micrOTOF-QII (Bruker Daltonics, Bremen, Germany)^13^. The MS data were analyzed using Data Analysis 4.0 software (Bruker Daltonics).

The lyophilized reaction product samples of PA-sugar chain and *p*NP were dissolved in dH_2_O and digested with either β-*N*-Acetylglucosaminidase from *Streptococcus pneumoniae* (Sigma), α-*N*-Acetylgalactosaminidase (NEB, Beverly, MA), or β-*N*-Acetylgalactosaminidase^56,57^ following the manufacturer’s protocols. The digested samples were analyzed by SF- or RP-HPLC.

### Determination of kinetic parameters

Kinetic parameters of BmGalNAcT were determined under standard reaction condition with varying the concentrations of UDP-GalNAc and GNM3A. The velocities under the various concentrations of substrates were measured by the time-dependent changes of the reaction products, then reaction products were analyzed as described above. The velocities versus the corresponding UDP-GalNAc or GNM3A concentrations were plotted and determined using a nonlinear regression analysis program of SigmaPlot software (Systat Software Inc., San Jose, CA).

### Preparation and structural analysis of neutral *N*-glycan

The Sf9 cells expressing BmGalNAcT were defatted with acetone and completely dried out. The sugar chains were released from crude glycoproteins by hydrazinolysis at 100 °C for 10 h. After *N*-acetylation of the hydrazinolysate with saturated sodium bicarbonate and acetic anhydride, the *N*-acetylated hydrazinolysate was desalted with Dowex 50 × 2 (Muromachi Kagaku Kogyo Kaisha), and fractionated on a TSK gel Toyopearl HW-40 (Tosoh) column (2.5 × 30 cm) in 0.1 N ammonia. The released sugar chains were 2-aminopyridine (PA)-labeled, as described previously, followed by fractionation on a TSK gel Toyopearl HW-40 column (2.5 × 30 cm) in 0.1 N ammonia. The sugar chains were detected by RP-HPLC as described above.

The molecular masses of the PA-sugar chains and the number of their sugar moieties were estimated by LC–MS/MS using an Agilent Technologies 1200 series instrument (Agilent Technologies, Santa Clara, CA) equipped with HCT plus software (Bruker Daltonics) as previously reported^13^.

### *N*-Glycan modeling

*N*-Glycan structures of GN2M3F, GN3M3, and GN3M3 with bisect GlcNAc were from a 3D structure libraries on a GLYCAM server (http://glycam.org/). Figures were prepared using PyMOL Molecular Graphics System, Version 1.7.1.1. (http://www.pymol.org/).

## Supplementary Information

Supplementary Information 1.

Supplementary Information 2.

Supplementary Information 3.

Supplementary Information 4.

Supplementary Information 5.

## References

[1] Varki A (1993). Biological roles of oligosaccharides: All of the theories are correct. Glycobiology.

[2] Helenius A, Aebi M (2001). Intracellular function of N-Linked Glycans. Science.

[3] Hebert DN, Lamriben L, Powers ET, Kelly JW (2014). The intrinsic and extrinsic effects of N-linked glycans on glycoproteostasis. Nat. Chem. Biol..

[4] Varki A (2017). Biological roles of glycans. Glycobiology.

[5] Wilson IB (2002). Glycosylation of proteins in plants and invertebrates. Curr. Opin. Struct. Biol..

[6] Shi X, Jarvis DL (2007). Protein *N*-glycosylation in the baculovirus-insect cell system. Curr. Drug Targets..

[7] Strasser R (2016). Plant protein glycosylation. Glycobiology.

[8] Tjondro HC, Loke I, Chatterjee S, Thaysen-Andersen M (2019). Human protein paucimannosylation: Cues from the eukaryotic kingdoms. Biol. Rev. Camb. Philos. Soc..

[9] Kubelka V (1993). Primary structures of the N-linked carbohydrate chains from honeybee venom phospholipase A_2_. Eur. J. Biochem..

[10] Staudacher E, März L (1998). Strict order of (Fuc to Asn-linked GlcNAc) fucosyltransferases forming core-difucosylated structures. Glycoconj. J..

[11] Paschinger K, Staudacher E, Stemmer U, Fabini G, Wilson IB (2007). Fucosyltransferase substrate specificity and the order of fucosylation in invertebrates. Glycobiology.

[12] Aoki K (2007). Dynamic developmental elaboration of *N*-linked glycan complexity in the *Drosophila melanogaster* embryo. J. Biol. Chem..

[13] Kajiura H, Hamaguchi Y, Mizushima H, Misaki R, Fujiyama K (2015). Sialylation potentials of the silkworm, *Bombyx mori*; *B. mori* possesses an active α2,6-sialyltransferase. Glycobiology.

[14] Krzewinski-Recchi MA (2003). Identification and functional expression of a second human β-galactoside α2,6-sialyltransferase, ST6Gal II. Eur. J. Biochem..

[15] Koles K, Irvine KD, Panin VM (2004). Functional characterization of Drosophila sialyltransferase. J. Biol. Chem..

[16] Kubelka V, Altmann F, März L (1995). The asparagine-linked carbohydrate of honeybee venom hyaluronidase. Glycoconj. J..

[17] Kimura M (2002). Occurrence of GalNAcβ1-4GlcNAc unit in N-glycan of royal jelly glycoprotein. Biosci. Biotechnol. Biochem..

[18] Aoki K, Tiemeyer M (2010). The glycomics of glycan glucuronylation in Drosophila melanogaster. Methods Enzymol..

[19] Stanton R (2017). The underestimated N-glycomes of lepidopteran species. Biochim. Biophys. Acta Gen. Subj..

[20] Kurz S (2015). Targeted release and fractionation reveal glucuronylated and sulphated N- and O-glycans in larvae of dipteran insects. J. Proteomics..

[21] Hykollari A, Malzl D, Stanton D, Eckmair B, Paschinger K (2019). Tissue-specific glycosylation in the honeybee: Analysis of the N-glycomes of *Apis mellifera* larvae and venom. Biochim. Biophys. Acta Gen. Subj..

[22] van Die I, van Tetering A, Bakker H, van den Eijnden DH, Joziasse DH (1996). Glycosylation in lepidopteran insect cells: Identification of a β1→4-*N*-acetylgalactosaminyltransferase involved in the synthesis of complex-type oligosaccharide chains. Glycobiology.

[23] Haines N, Irvine KD (2007). Functional roles for β1,4-N-acetlygalactosaminyltransferase-A in Drosophila larval neurons and muscles. Genetics.

[24] Vadaie N, Jarvis DL (2004). Molecular cloning and functional characterization of a Lepidopteran insect β4-*N*-acetylgalactosaminyltransferase with broad substrate specificity, a functional role in glycoprotein biosynthesis, and a potential functional role in glycolipid biosynthesis. J. Biol. Chem..

[25] Raman J, Guan Y, Perrine CL, Gerken TA, Tabak LA (2012). UDP-*N*-acetyl-α-d-galactosamine:polypeptide *N*-acetylgalactosaminyltransferases: completion of the family tree. Glycobiology.

[26] Ramakrishnan B, Qasba PK (2010). Structure-based evolutionary relationship of glycosyltransferases: A case study of vertebrate β1,4-galactosyltransferase, invertebrate β1,4-*N*-acetylgalactosaminyltransferase and α-polypeptidyl-*N*-acetylgalactosaminyltransferase. Curr. Opin. Struct. Biol..

[27] Ramakrishnan B, Qasba PK (2007). Role of a single amino acid in the evolution of glycans of invertebrates and vertebrates. J. Mol. Biol..

[28] Asano M (1997). Growth retardation and early death of β-1,4-galactosyltransferase knockout mice with augmented proliferation and abnormal differentiation of epithelial cells. EMBO J..

[29] Lu Q, Hasty P, Shur BD (1997). Targeted mutation in β1,4-galactosyltransferase leads to pituitary insufficiency and neonatal lethality. Dev. Biol..

[30] Hansske B (2002). Deficiency of UDP-galactose:*N*-acetylglucosamine β-1,4-galactosyltransferase I causes the congenital disorder of glycosylation type IId. J. Clin. Investig..

[31] Haines N, Irvine KD (2005). Functional analysis of Drosophila β1,4-*N*-acetlygalactosaminyltransferases. Glycobiology.

[32] Veyhl J (2017). The directed migration of gonadal distal tip cells in *Caenorhabditis elegans* requires NGAT-1, a β1,4-N-acetylgalactosaminyltransferase enzyme. PLoS ONE.

[33] Shimomura M (2009). KAIKObase: An integrated silkworm genome database and data mining tool. BMC Genom..

[34] Miyazaki T (2019). Biochemical characterization and mutational analysis of silkworm *Bombyx mori* β-1,4-*N*-acetylgalactosaminyltransferase and insight into the substrate specificity of β-1,4-galactosyltransferase family enzymes. Insect Biochem. Mol. Biol..

[35] Ramakrishnan B, Boeggeman E, Ramasamy V, Qasba PK (2004). Structure and catalytic cycle of β-1,4-galactosyltransferase. Curr. Opin. Struct. Biol..

[36] Togayachi A, Sato T, Narimatsu H (2006). Comprehensive enzymatic characterization of glycosyltransferases with a β3GT or β4GT motif. Methods Enzymol..

[37] Qasba PK, Ramakrishnan B, Boeggeman E (2008). Structure and function of β-1,4-galactosyltransferase. Curr. Drug Targets.

[38] Harvey DJ (2005). Fragmentation of negative ions from carbohydrates: part 3. Fragmentation of hybrid and complex N-linked glycans. J. Am. Soc. Mass Spectrom..

[39] Wuhrer M (2013). Glycomics using mass spectrometry. Glycoconj. J..

[40] Dojima T (2009). Comparison of the N-linked glycosylation of human β1,3-*N*-acetylglucosaminyltransferase 2 expressed in insect cells and silkworm larvae. J. Biotechnol..

[41] Dell A (1995). Structural analysis of the oligosaccharides derived from glycodelin, a human glycoprotein with potent immunosuppressive and contraceptive activities. J. Biol. Chem..

[42] Seppo A, Moreland M, Schweingruber H, Tiemeyer M (2000). Zwitterionic and acidic glycosphingolipids of the *Drosophila melanogaster* embryo. Eur. J. Biochem..

[43] Palcic MM, Hindsgaul O (1991). Flexibility in the donor substrate specificity of β1,4-galactosyltransferase: Application in the synthesis of complex carbohydrates. Glycobiology.

[44] Tomiya N, Ailor E, Lawrence SM, Betenbaugh MJ, Lee YC (2001). Determination of nucleotides and sugar nucleotides involved in protein glycosylation by high-performance anion-exchange chromatography: Sugar nucleotide contents in cultured insect cells and mammalian cells. Anal. Biochem..

[45] Chang KH (2007). Expression of recombinant cyclooxygenase 1 in *Drosophila melanogaster* S2 cells transformed with human β1,4-galactosyltransferase and Galβ1,4-GlcNAc α2,6-sialyltransferase. Biotechnol. Lett..

[46] Kim YK (2011). Expression of β-1,4-galactosyltransferase and suppression of β-*N*-acetylglucosaminidase to aid synthesis of complex *N*-glycans in insect *Drosophila* S2 cells. J. Biotechnol..

[47] Geisler C, Mabashi-Asazuma H, Kuo CW, Khoo KH, Jarvis DL (2015). Engineering β1,4-galactosyltransferase I to reduce secretion and enhance *N*-glycan elongation in insect cells. J. Biotechnol..

[48] Tomono T, Kojima H, Fukuchi S, Tohsato Y, Ito M (2015). Investigation of glycan evolution based on a comprehensive analysis of glycosyltransferases using phylogenetic profiling. Biophys. Physicobiol..

[49] Kawar ZS, Haslam SM, Morris HR, Dell A, Cummings RD (2005). Novel poly-GalNAcβ1-4GlcNAc (LacdiNAc) and fucosylated poly-LacdiNAc *N*-glycans from mammalian cells expressing β1,4-*N*-acetylgalactosaminyltransferase and α1,3-fucosyltransferase. J. Biol. Chem..

[50] Sanders RD, Sefton JM, Moberg KH, Fridovich-Keil JL (2010). UDP-galactose 4' epimerase (GALE) is essential for development of *Drosophila melanogaster*. Dis. Model Mech..

[51] Haines N, Stewart BA (2007). Functional roles for β1,4-*N*-acetlygalactosaminyltransferase-A in Drosophila larval neurons and muscles. Genetics.

[52] Takasu Y (2010). Targeted mutagenesis in the silkworm *Bombyx mori* using zinc finger nuclease mRNA injection. Insect Biochem. Mol. Biol..

[53] Sajwan S (2013). Efficient disruption of endogenous Bombyx gene by TAL effector nucleases. Insect Biochem. Mol. Biol..

[54] Tsubota T, Sezutsu H (2017). Genome editing of silkworms. Methods Mol. Biol..

[55] Ma SY, Smagghe G, Xia QY (2019). Genome editing in *Bombyx mori*: New opportunities for silkworm functional genomics and the sericulture industry. Insect Sci..

[56] Tanaka A, Ozaki S (1997). Purification and characterization of β-N-acetylgalactosaminidase from *Bacillus* sp. AT173-1. J. Biochem..

[57] Kimura Y (2006). Tumor antigen occurs in *N*-glycan of royal jelly glycoproteins: Honeybee cells synthesize T-antigen unit in *N*-glycan moiety. Biosci. Biotechnol. Biochem..

