# Supplementary material for: Bombyx mori β1,4-N-acetylgalactosaminyltransferase possesses relaxed donor substrate specificity in N-glycan synthesis
Source: Sci Rep. 2021 Mar 9;11:5505. doi: 10.1038/s41598-021-84771-z (PMC7943597; doi:10.1038/s41598-021-84771-z)
Supplement: Supplementary file 1 — Supplementary Information 1. [file 41598_2021_84771_MOESM1_ESM.docx]

**Description of Additional Supplementary Files**

**File Name: Supplementary Data 1**

**Description:**

• **Supplementary Data 1:** The original, unprocessed versions of Figures used in Supplementary Figure S3. PCR products of *Bmrp49* with different cycle number were shown.

• **Supplementary Data 2:** The original, unprocessed versions of Figures used in Figure 2a and 2b.

• **Supplementary Data 3:** The original, unprocessed versions of Figure used in Supplementary Figure S12.
