# Supplementary material for: Bombyx mori β1,4-N-acetylgalactosaminyltransferase possesses relaxed donor substrate specificity in N-glycan synthesis
Source: Sci Rep. 2021 Mar 9;11:5505. doi: 10.1038/s41598-021-84771-z (PMC7943597; doi:10.1038/s41598-021-84771-z)
Supplement: Supplementary file 2 — Supplementary Information 2. [file 41598_2021_84771_MOESM2_ESM.pptx]

## Slide 1
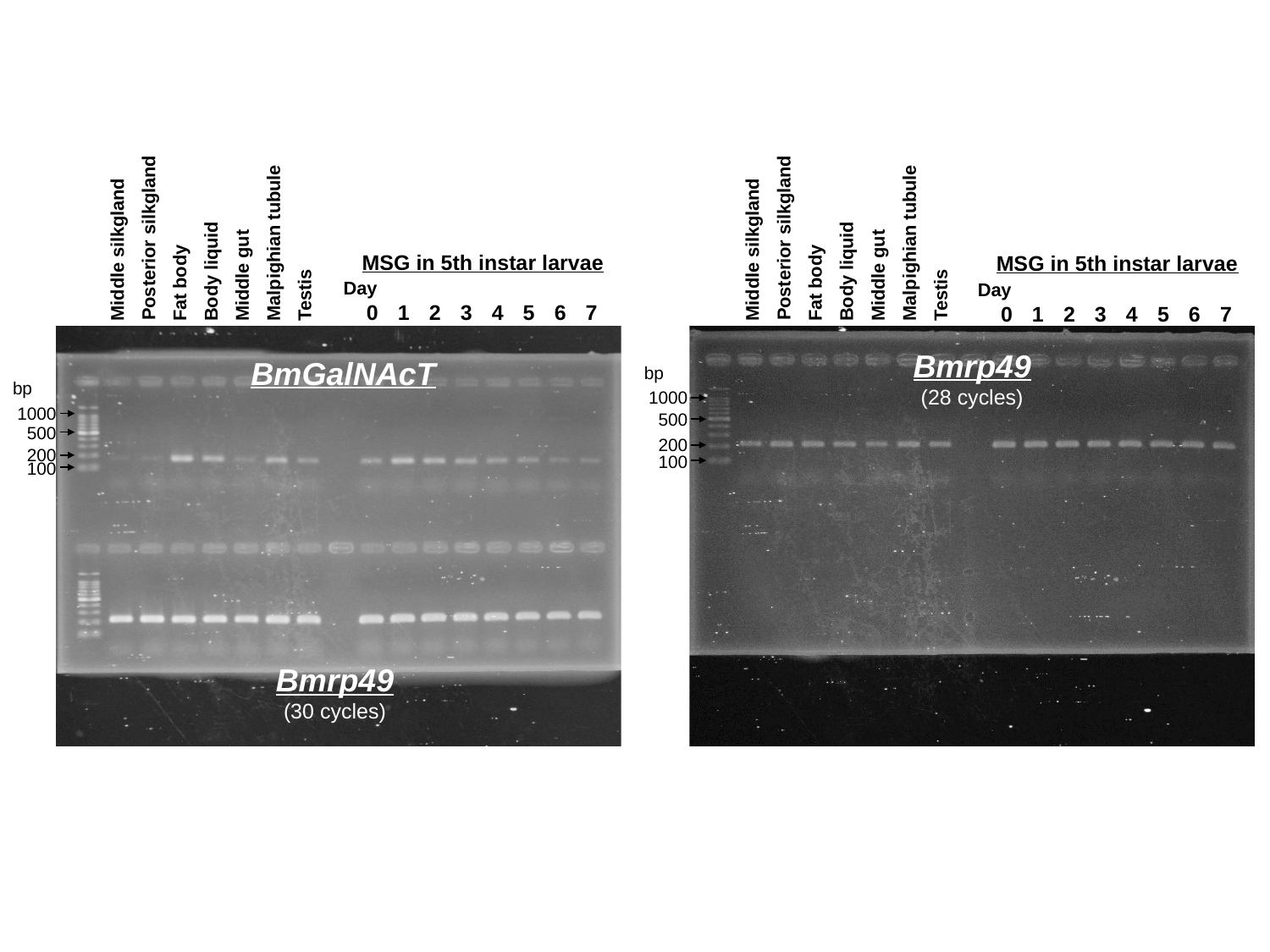

Posterior silkgland
Posterior silkgland
Malpighian tubule
Malpighian tubule
Middle silkgland
Middle silkgland
MSG in 5th instar larvae
MSG in 5th instar larvae
Body liquid
Body liquid
Middle gut
Middle gut
Fat body
Fat body
Day
Day
Testis
Testis
0
1
2
3
4
5
6
7
0
1
2
3
4
5
6
7
Bmrp49
(28 cycles)
BmGalNAcT
bp
1000
bp
1000
500
500
200
200
100
100
Bmrp49
(30 cycles)
