# Supplementary figures and images for: Bombyx mori β1,4-N-acetylgalactosaminyltransferase possesses relaxed donor substrate specificity in N-glycan synthesis
Source: Sci Rep. 2021 Mar 9;11:5505. doi: 10.1038/s41598-021-84771-z (PMC7943597; doi:10.1038/s41598-021-84771-z)

## Slide 1
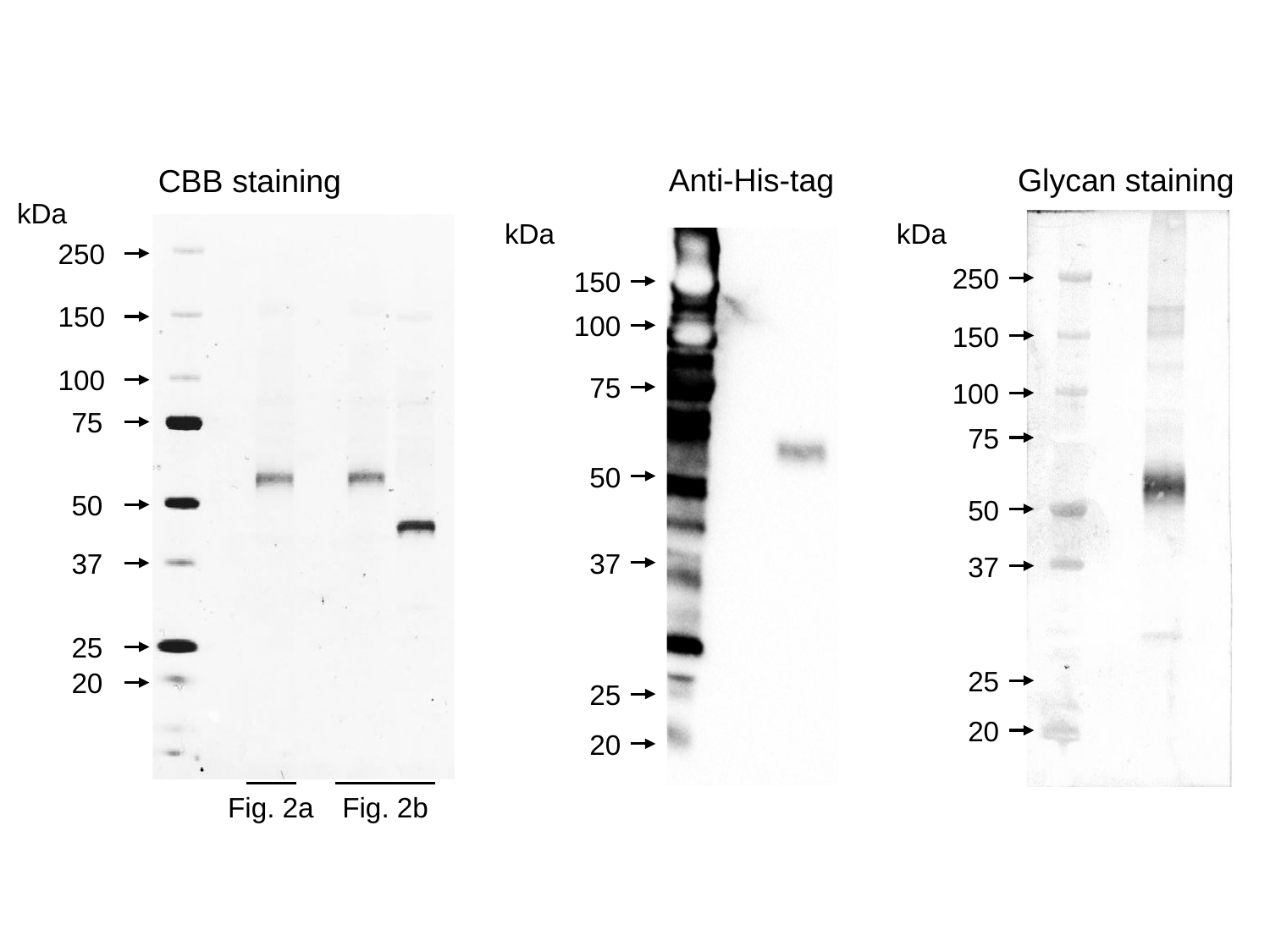

Anti-His-tag
Glycan staining
CBB staining
kDa
kDa
kDa
250
250
150
150
100
150
100
75
100
75
75
50
50
50
37
37
37
25
25
20
25
20
20
Fig. 2a
Fig. 2b

Supplement: Supplementary file 3 — Supplementary Information 3. [file 41598_2021_84771_MOESM3_ESM.pptx]

## Slide 1
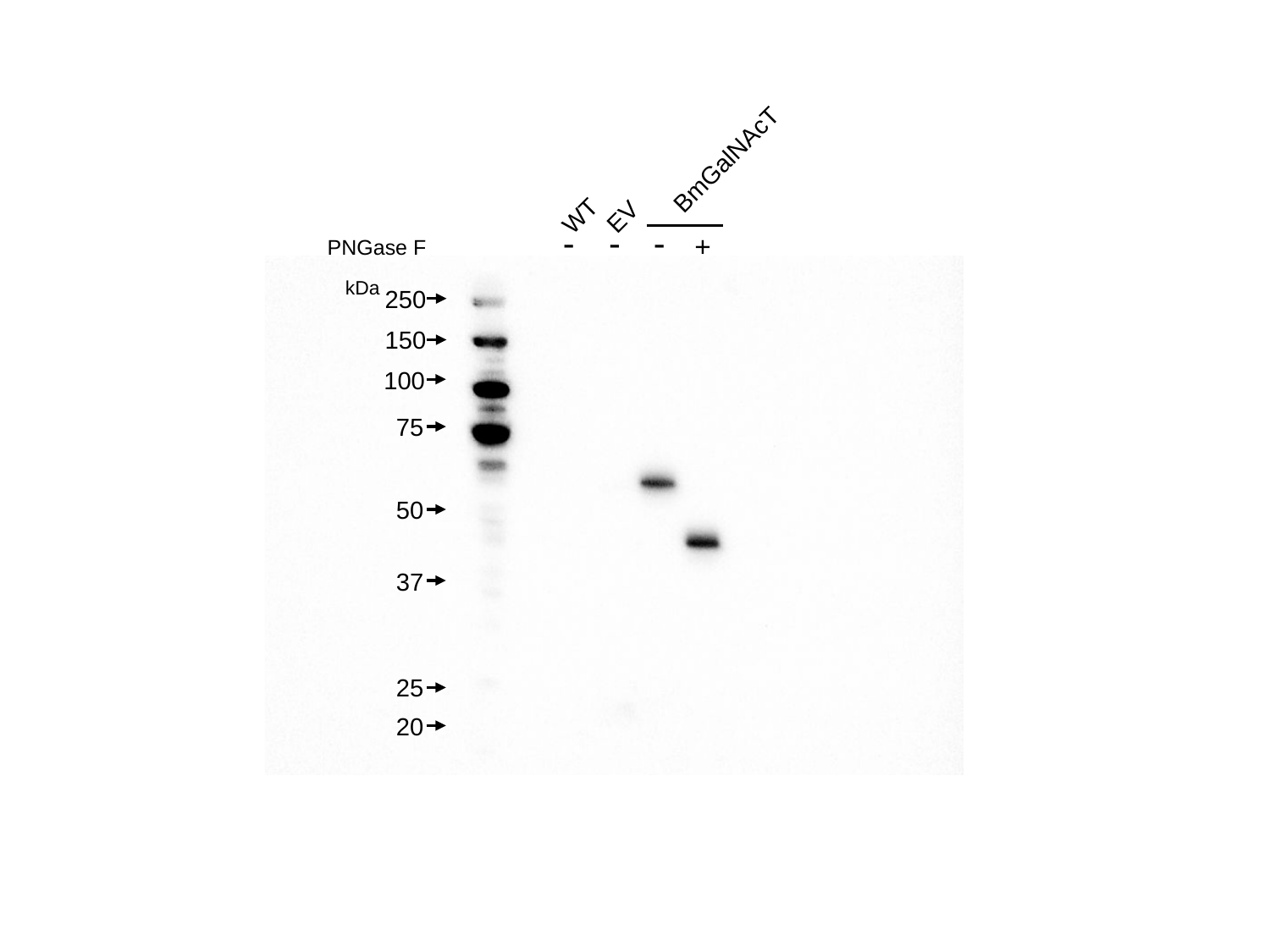

BmGalNAcT
WT
EV
-
-
-
+
PNGase F
kDa
250
150
100
75
50
37
25
20

Supplement: Supplementary file 4 — Supplementary Information 4. [file 41598_2021_84771_MOESM4_ESM.pptx]
