# Supplementary material for: Bombyx mori β1,4-N-acetylgalactosaminyltransferase possesses relaxed donor substrate specificity in N-glycan synthesis
Source: Sci Rep. 2021 Mar 9;11:5505. doi: 10.1038/s41598-021-84771-z (PMC7943597; doi:10.1038/s41598-021-84771-z)
Supplement: Supplementary file 5 — Supplementary Information 5. [file 41598_2021_84771_MOESM5_ESM.pdf]

Bombyx mori β1,4-_N_-acetylgalactosaminyltransferase possesses relaxed donor substrate specificity in _N_-glycan synthesis

Supplementary Information

Hiroyuki Kajiura, Ryousuke Miyauchi, Akemi Kakudo, Takao Ohashi, Ryo Misaki & Kazuhito Fujiyama*

International Center for Biotechnology, Osaka University, 2-1 Yamada-oka, Suita-shi, Osaka, 565-0871, Japan

*Correspondence should be addressed to K.F. (fujiyama@icb.osaka-u.ac.jp)

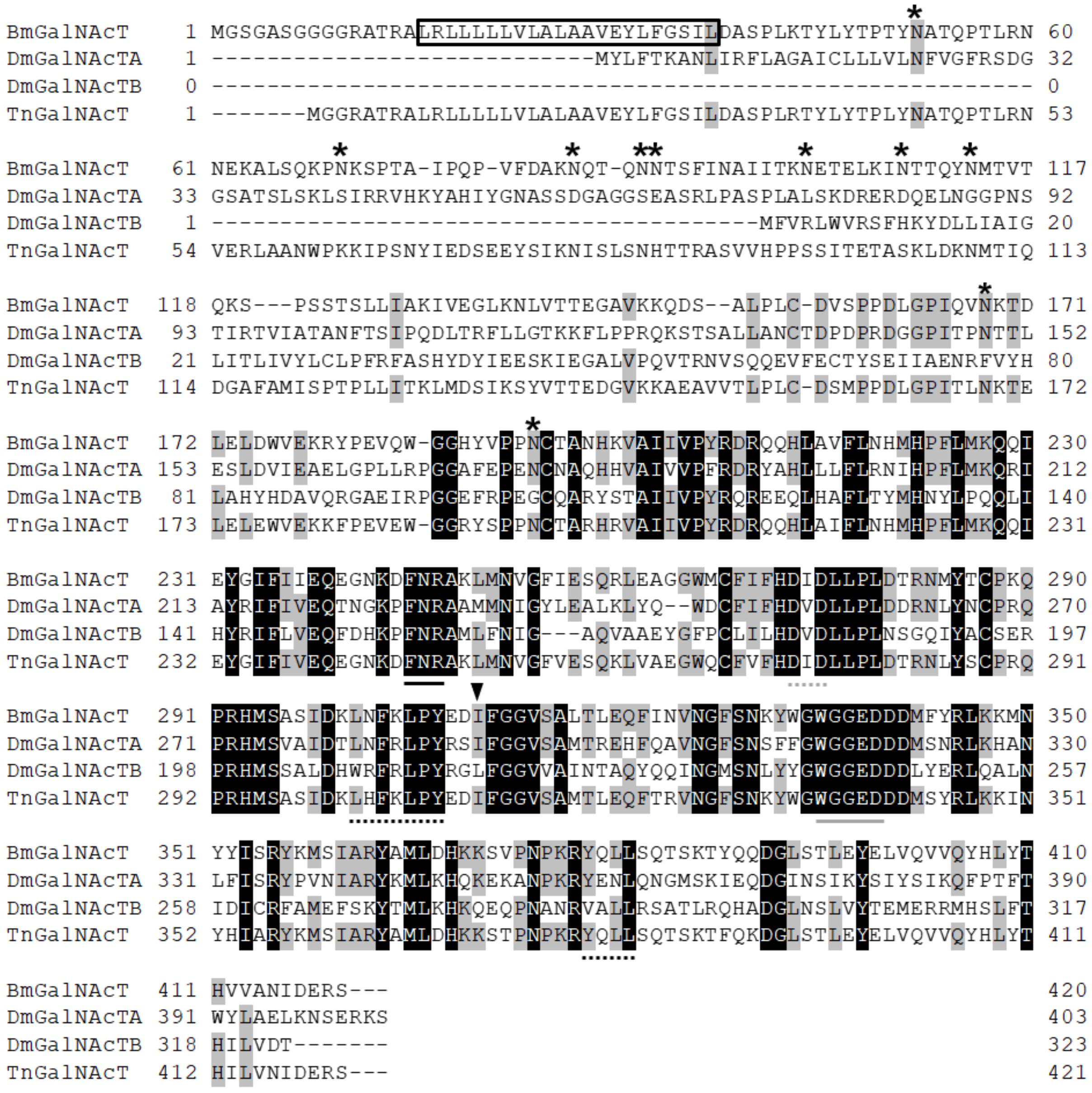

Supplementary Figure S1 Alignment of amino acid sequences of BmGalNAcT and insect β1,4-GalNAcTs. Identical and similar sequences are shaded in black and gray, respectively. The black box indicates the putative transmembrane region and asterisks provide the potential *N*-glycosylation site, N-X-S/T. The black triangle shows the amino acid determinant of activity defined by Ramakrishnan and Qasba (2007). Conserved sequences in GT family 7 proteins, the donor substrate binding site, the acceptor substrate binding site, the site binding both the donor and acceptor substrates, and the ion binding site are indicated by underlining with black, black dotted, gray, and gray dotted lines.

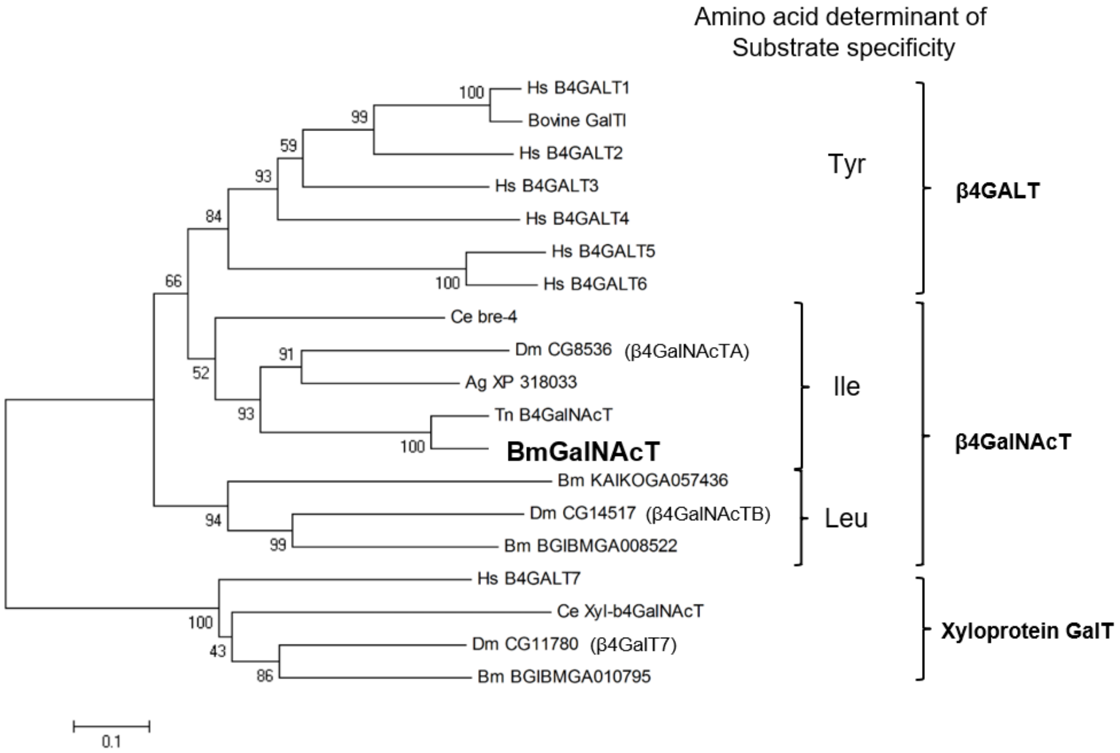

Supplementary Figure S2 Phylogenetic analysis of BmGalNAcT, invertebrate GalNAcTs, and human β1,4-galactosyltransferases.

The phylogenetic tree was constructed using CLUSTALW (http://www.genome.jp/tools/clustalw/). The key amino acid determinants of GT7 family proteins are shown. BmGalNAcT is shown in bold. The *scale bar* shows relative units of evolutionary distances.

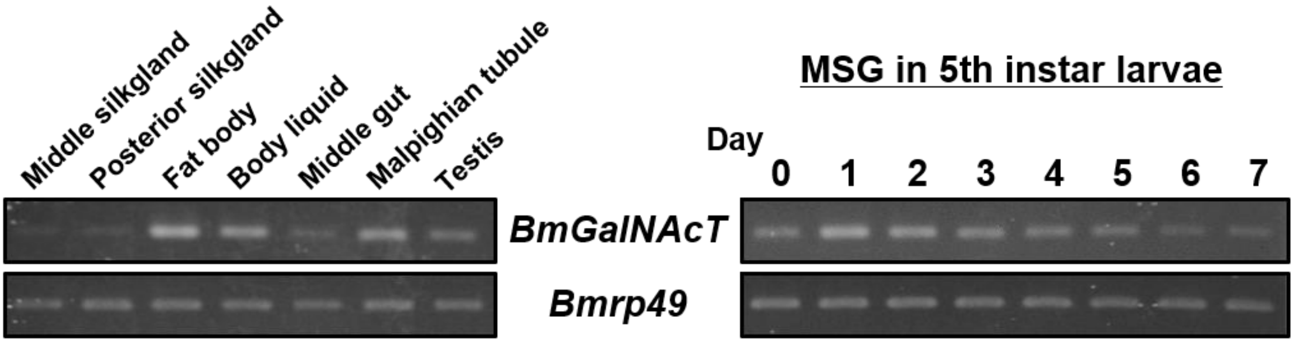

Supplementary Figure S3 Expression profile of BmGalNAcT.

Expression levels of *BmGalNAcT* in different organs and in the MSG developmental stages of 5th instar larvae were analyzed by quantitative RP-PCR analysis. *Bmrp49* is used as a control for the mRNA expressions of each organ and stage.

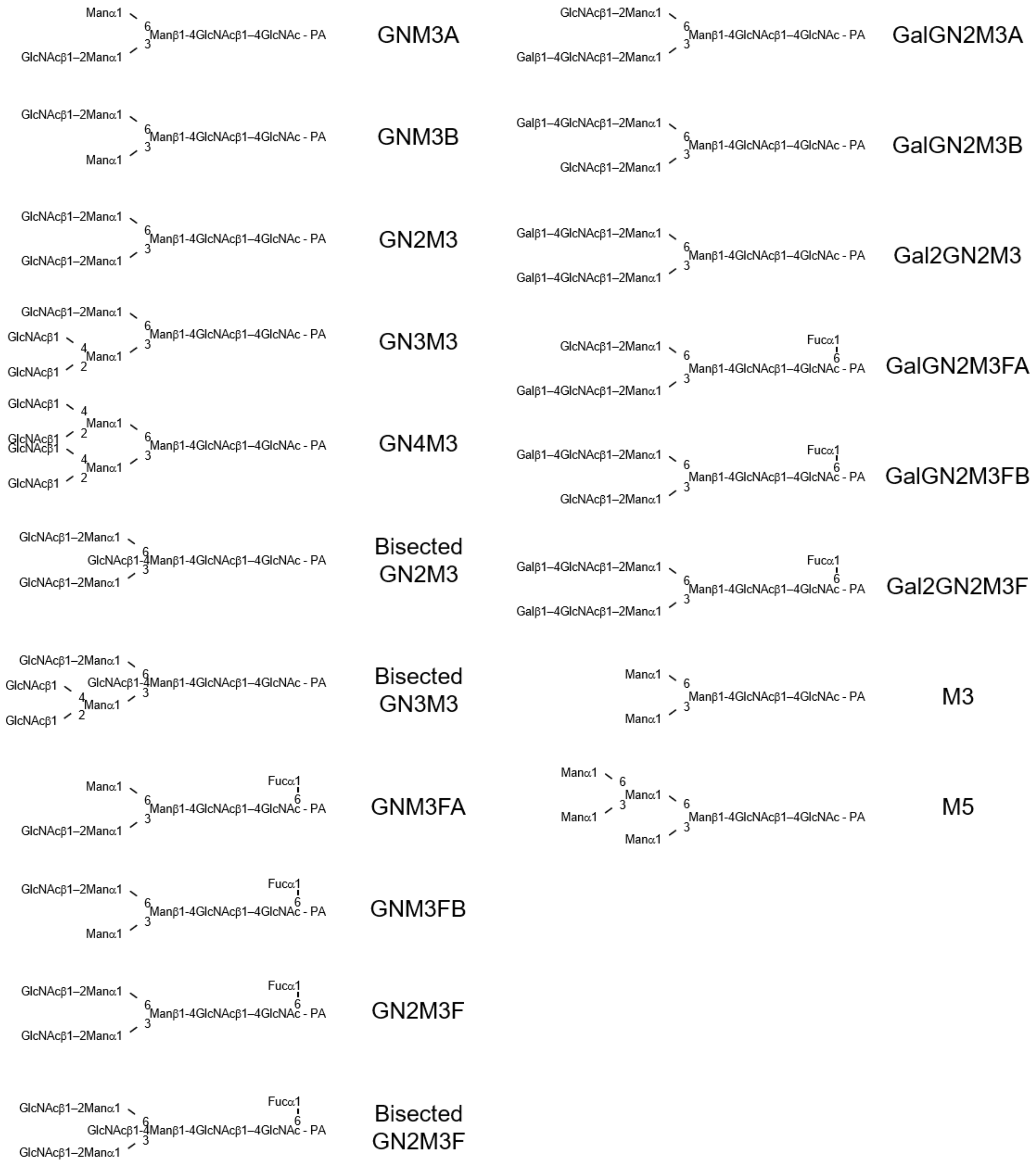

Supplementary Figure S4 PA-sugar chain structures used for substrate specificity analysis of BmGalNAcT.

Man, Mannose; GlcNAc, *N*-Acetylglucosamine; Fuc, Fucose; Gal, Galactose.

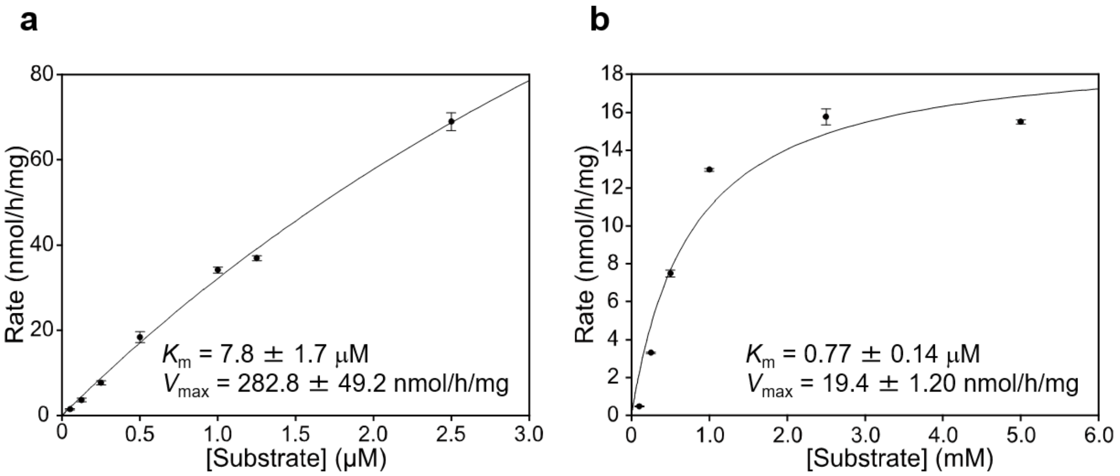

Supplementary Figure S5 Determination of kinetic parameters of BmGalNAcT.

K_m and V_{max} were determined using by plotting the velocity versus the corresponding (a) GNM3A or (b) UDP-GalNAc concentrations using a nonlinear regression analysis program of SigmaPlot software (Systat Software Inc., San Jose, CA).

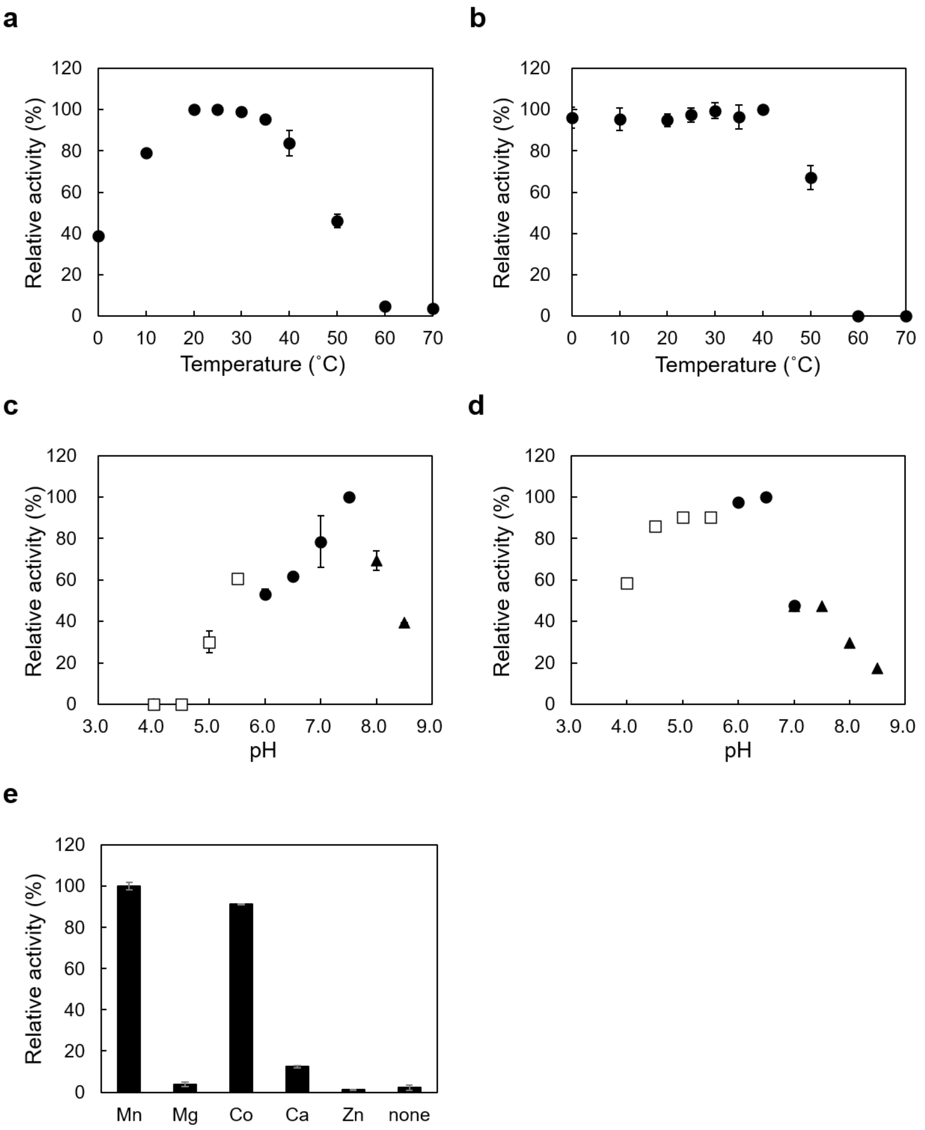

Supplementary Figure S6 Enzymatic properties of BmGalNAcT.

(a) Optimum temperature, (b) temperature stability (c) pH dependency, (d) pH stability, and (e) metal-ion dependency were analyzed. The enzyme reactions were performed at 0, 10, 20, 25, 30, 35, 40, 50, 60, and 70°C in optimum temperature analysis using GNM3A as the substrate and pH dependency was measured using sodium acetate buffer (pH 4.0-5.5), open square; cacodylic acid buffer (pH 6.0-7.5), black circle; and Tris-HCl (pH 8.0 and 8.5), black triangle. Fort the determination of temperature, For the temperature stability, the enzyme solution was incubated at various temperatures for 1 h and then cooled on ice for 5 min. The activity assay was performed under standard conditions. For the pH stabilities, the enzyme solution was incubated at various pHs with 20 mM buffer in 10 μl for 1 h. Then, the enzymatic reactions were initiated by the addition of 500 mM cacodylic acid buffer pH 8.0, Mn^{2+}, and GNM3A as the substrate in final volume of 100 μl. The reaction products were analyzed by HPLC and the results are presented as% relative activity against the highest sample. To determine the effect of metal ions, the reaction was performed as described previously (Kajiura *et al.*, *Glycobiology* **20**, 235-247(2010)).

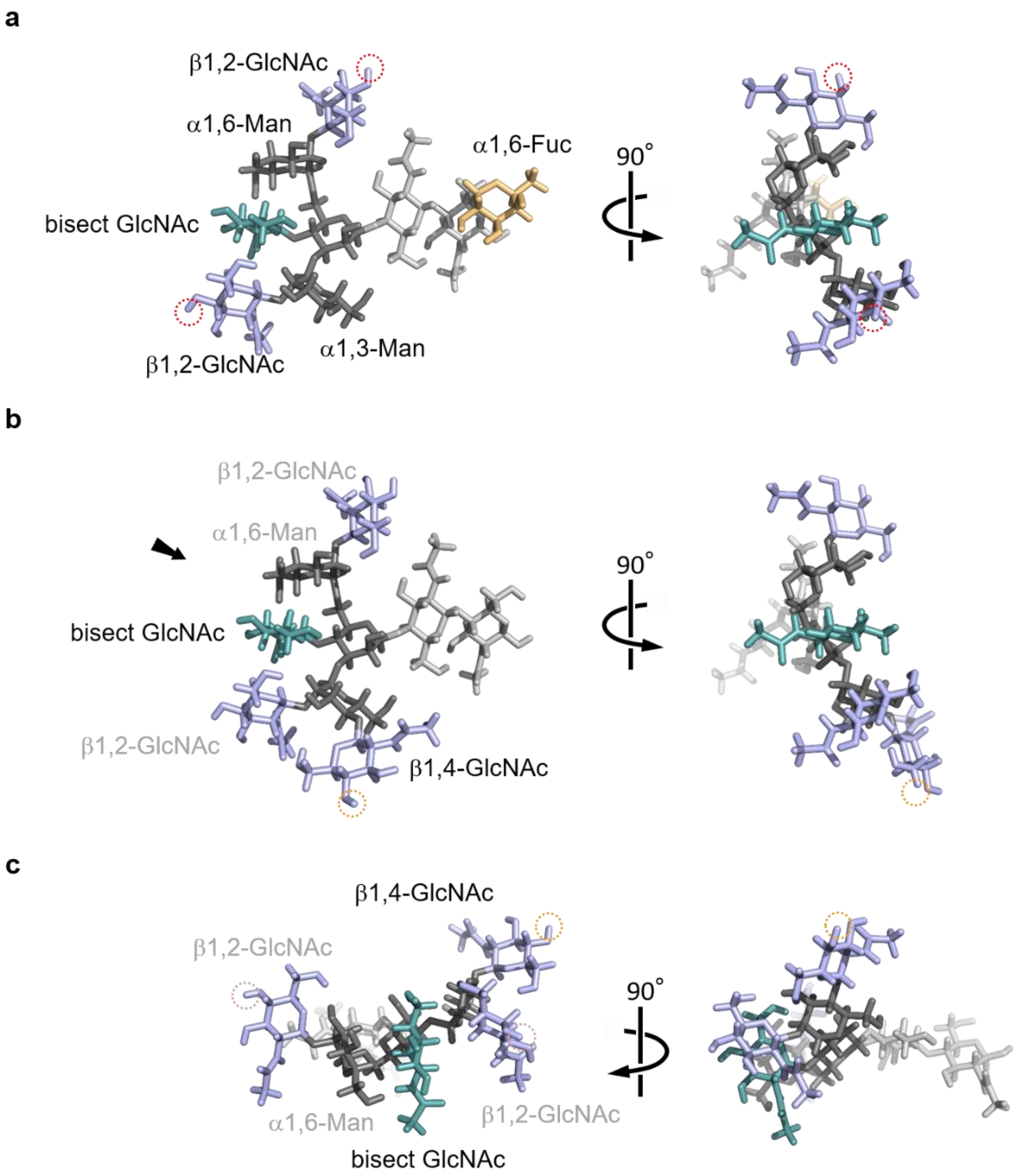

Supplementary Figure S7 Three-dimensional models of *N*-glycan structures.

(a) Front and side views of GN2M3F with bisected GlcNAc. The sugar residues indicated by blue, green, dark gray, light gray, and yellow indicate β1,2-GlcNAc, bisected GlcNAc, Man, core GlcNAc, and Fuc residues, respectively. A red-dotted circle represents the OH group at the C4-position of β1,2-GlcNAc residues used for GalNAc, Gal, and GlcNAc transfer.

(b) Front and side views of GN3M3 with bisected GlcNAc. An orange-dotted circle represents the OH group at the C4-position of β1,4-GlcNAc residues.

(c) Schematic representation of GN3M3 with bisected GlcNAc observed from the point of view indicated by the arrow in (b). The target OH group for GalNAc transfer at the C4-position of β1,4-GlcNAc is located above the face where β1,2-GlcNAc residues, α1,6-Man, and bisected GlcNAc are aligned.

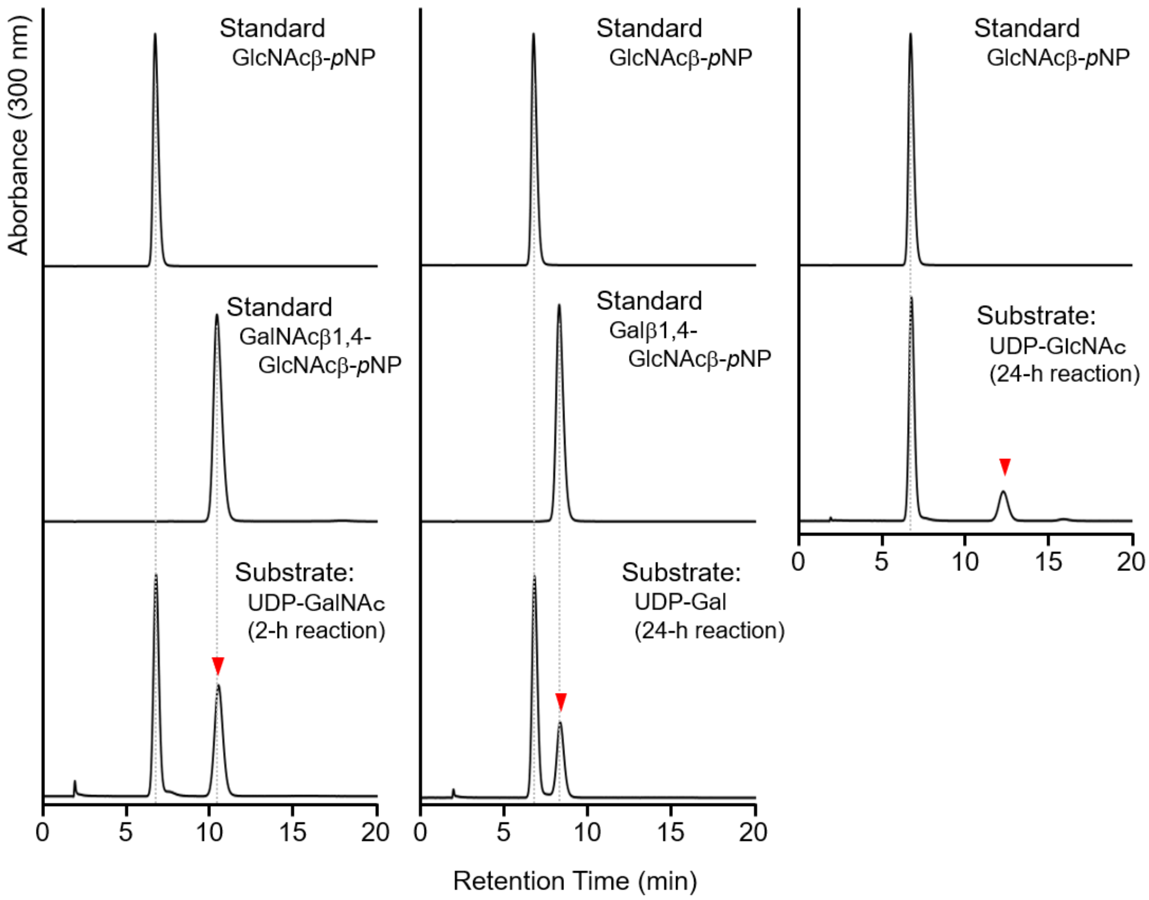

Supplementary Figure S8 Donor substrate specificity of BmGalNAcT.

The reaction products of BmGalNAcT using UDP-GalNAc (2 h), UDP-Gal (24 h), and UDP-GlcNAc (24 h) as donor substrates and GlcNAcβ-*p*NP as an acceptor substrate were separated by RP-HPLC. The red triangles indicate the reaction products.

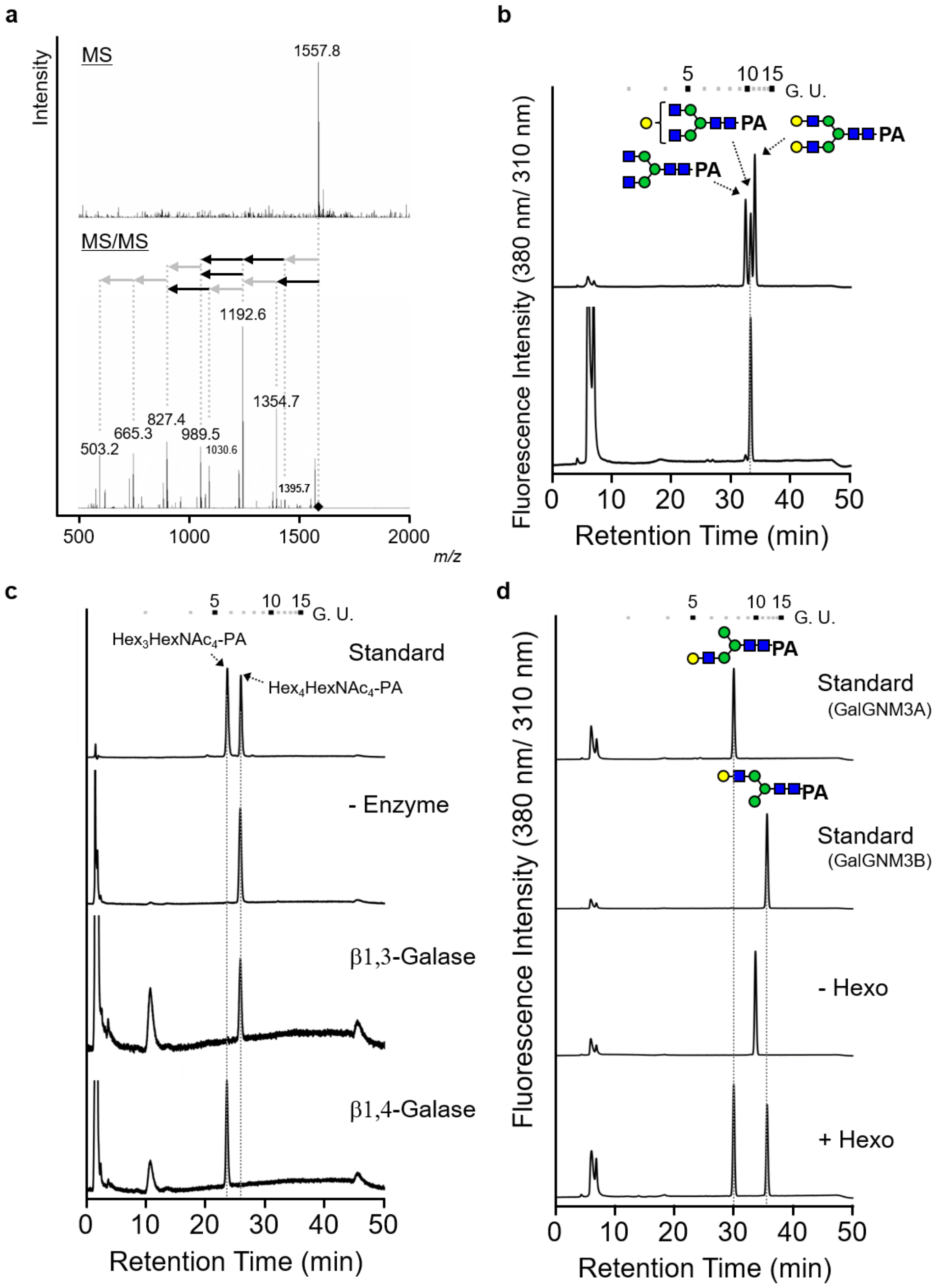

Supplementary Figure S9 Structural determination of galactosylated PA-sugar chain, peak a.

(a) MS and MS/MS analysis of peak a. The mass of the precursor ion, *m/z* 1557.8, corresponded to $HexNAc_2Hex_4HexNAc_2$-PA. The black and gray arrows represent *N*-acetylhexosamine and hexose, respectively.

(b) RP-HPLC analysis of peak a. Upper and lower chromatographs of RP-HPLC show the PA-sugar chains of standards and peak a. Green and yellow circles and blue boxes indicate Man, Gal, and GlcNAc.

(c) Linkage analysis of the terminal galactose residue of Peak a by linkage-specific galactosidase digestion. Peak a was digested by β1,3-galactosidase or β1,4-galactosidase, followed by comparison with the authentic PA-sugar chain in SF-HPLC.

(d) Structural assignment of peak a by hexosaminidase (HEXO) digestion and comparison with the authentic PA-sugar chain in RP-HPLC. Numbers at the top represent the elution positions of glucose units.

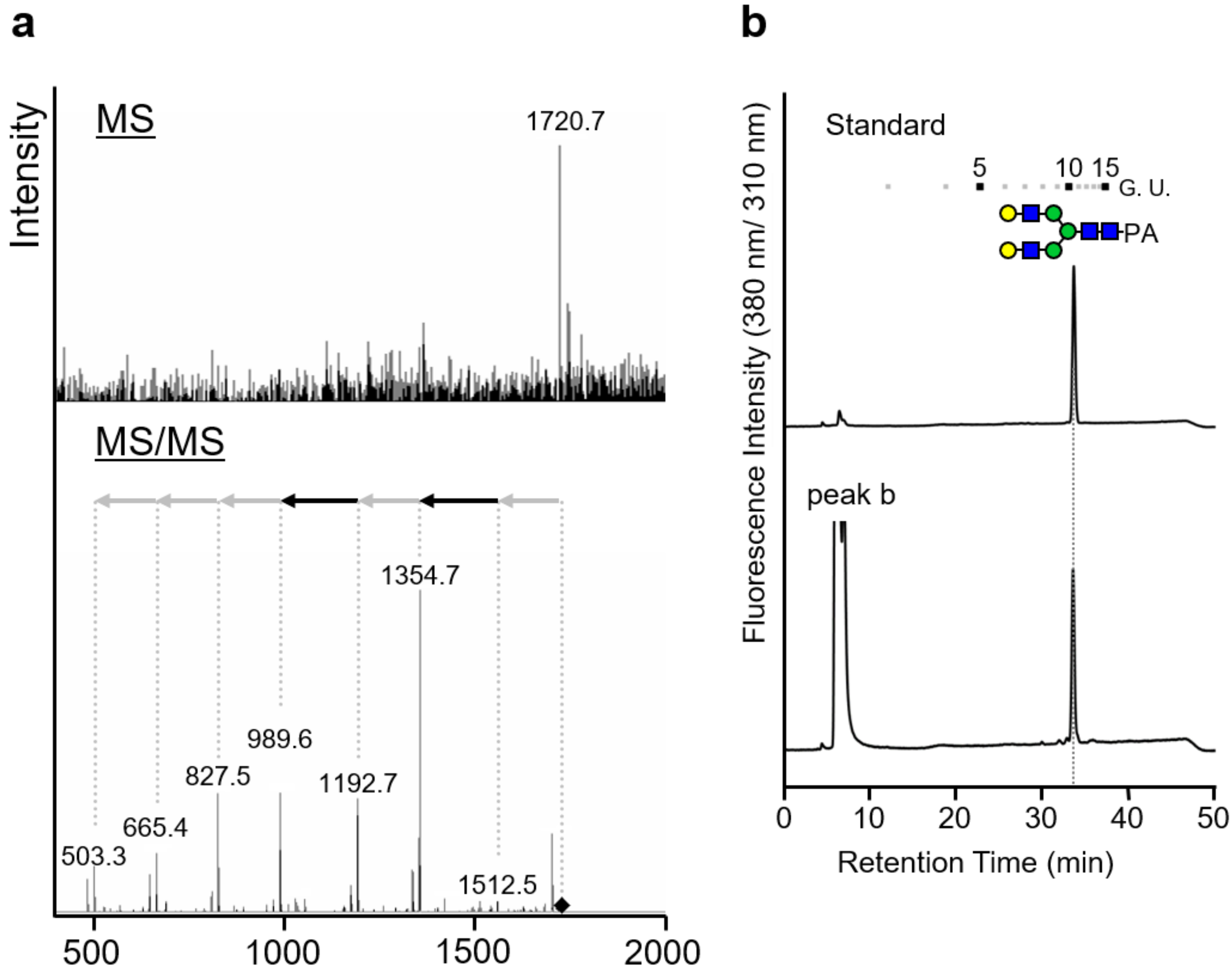

Supplementary Figure S10 Structural determination of the bi-galactosylated product of BmGalNAcT.
(a) MS and MS/MS analysis of peak b in Fig. 4. The mass of the precursor ion, *m/z* 1720.7, corresponded to $HexNAc_2Hex_5HexNAc_2$-PA. The black and gray arrows represent *N*-acetylhexosamine and hexose, respectively. (b) RP-HPLC analysis of peak b. Upper and lower chromatographs show PA-sugar chains of standards and peak b. Green and yellow circles and blue boxes indicate Man, Gal, and GlcNAc. Numbers at the top represent the elution positions of glucose units on the basis of the elution times of PA-isomalto-oligosaccharides with degrees of polymerization from 3 to 15.

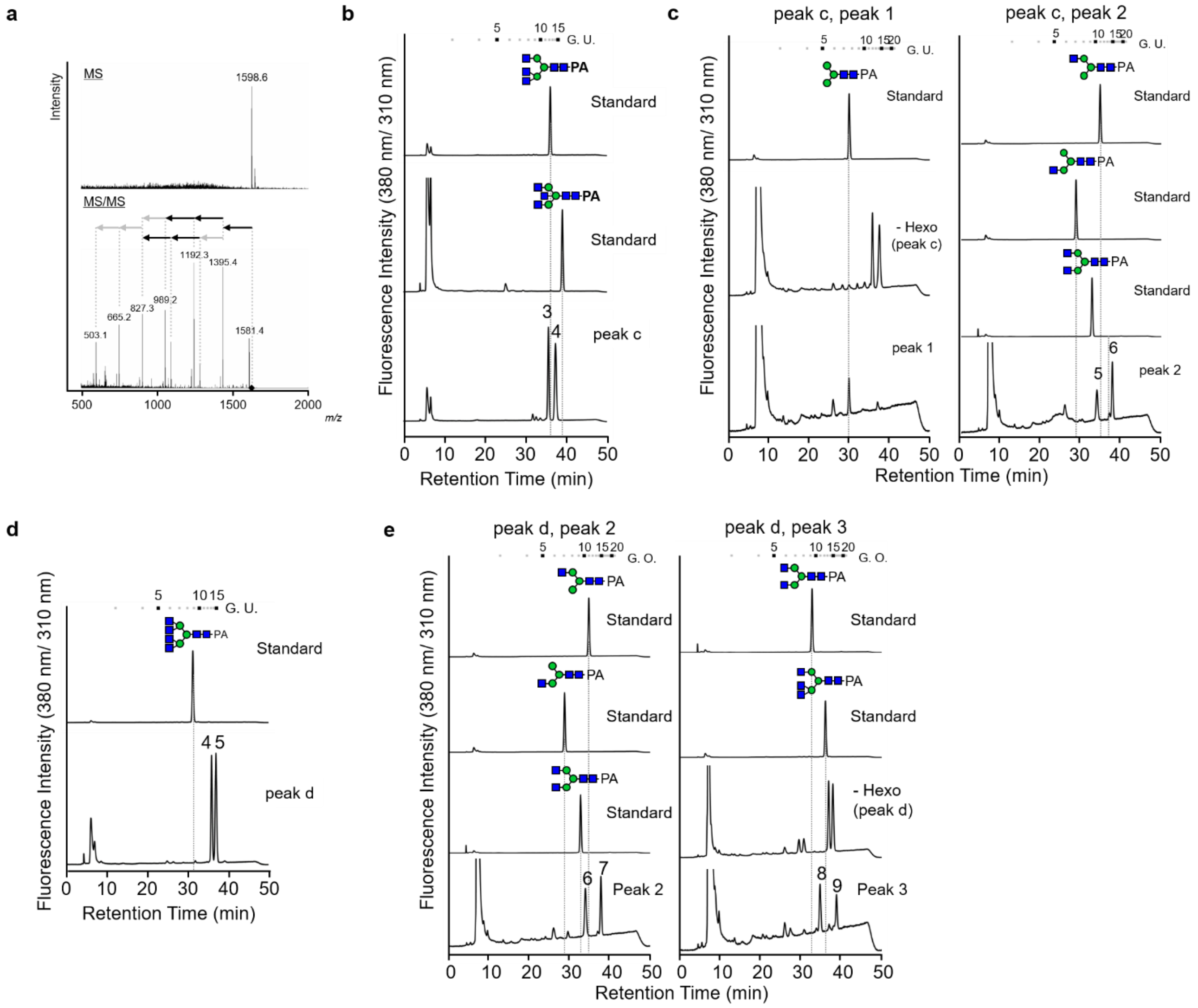

Supplementary Figure S11 Structural determination of *N*-acetylglucosaminylated sugar chains.

(a) MS and MS/MS analysis of peak c. The mass of the precursor ion, *m/z* 1598.6, corresponded to $HexNAc_3Hex_3HexNAc_2$-PA. A diamond represents the precursor ion. The black and gray arrows represent *N*-acetylhexosamine and hexose, respectively.

(b) RP-HPLC analysis of peak c. Peak c did not correspond to authentic PA-sugar chains tri-antenna sugar chains and bisected-GlcNAc-carrying sugar chains.

(c) RP-HPLC analysis of *N*-acetylhexosaminidase-digested peak c. The elution positions of *N*-acetylhexosaminidase-digested products were compared with those of authentic PA-sugar chains. Numbers at the top represent the elution positions of glucose units with degrees of polymerization from 3 to 20.

(d) RP-HPLC analysis of peak d. Peak d did not correspond to the authentic PA-sugar chains tetra-antenna sugar chain.

(e) RP-HPLC analysis of *N*-acetylhexosaminidase-digested peak d.

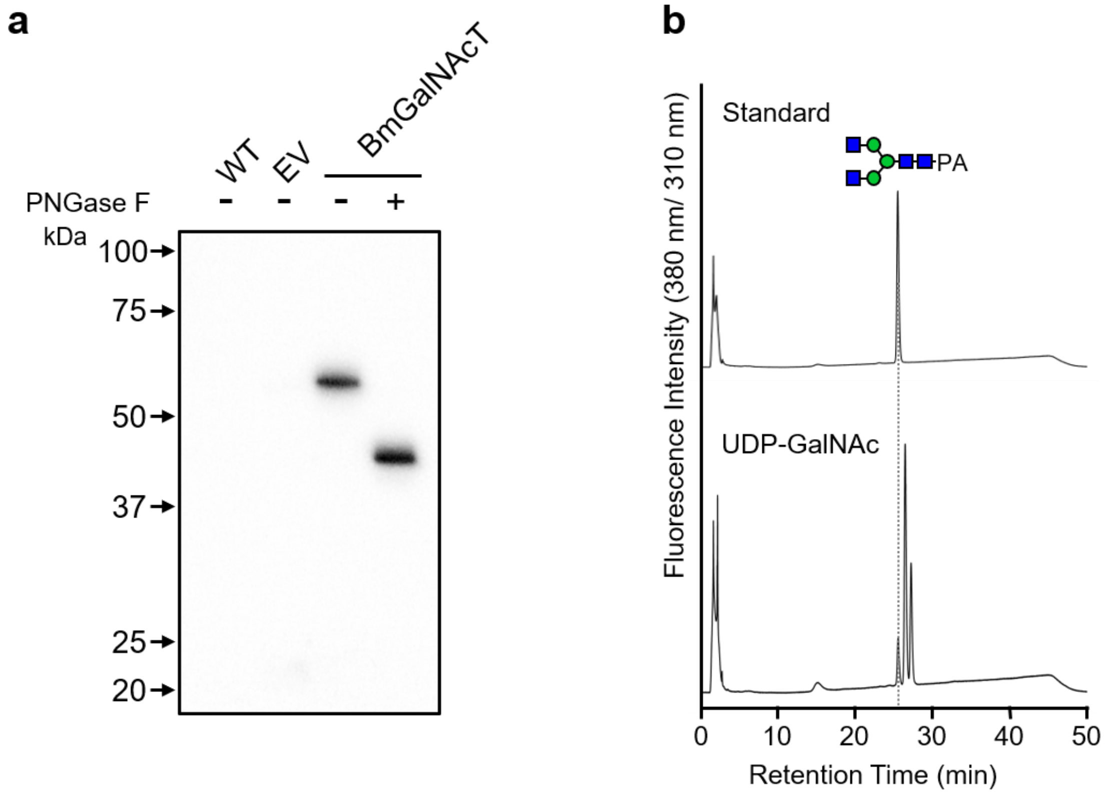

Supplementary Figure S12 Expression of BmGalNAcT in Sf9 cells for *in vivo* β1,4-*N*-acetylgalactosaminylation.

(a) Detection of full length BmGalNAcT with C-terminal His-tag expressed in Sf9 cells. Purified BmGalNAcT was further digested by PNGase F to de-glycosylate recombinant BmGalNAcT. BmGalNAcTs without and with PNGase F digestion were detected by western blotting using anti-His antibody. EV: Empty vector.

(b) RP-HPLC analysis of reaction products. BmGalNAcT reaction was carried out using UDP-GalNAc and GN2M3 as a donor and an acceptor substrate, respectively.

Supplementary Table S1 Details of *N*-glycans detected in BmGalNAcT expressing Sf9 cells

Structure		WT		BmGalNAcT	
		m/z $[M+H]^+$	Ratio (%)	*m/z* $[M+H]^+$	Ratio (%)
Mannose type *N*-glycan					
	M3	989.6	0.7	990.5	0.9
	M4	1153.6	0.5	1153.6	0.4
	M5	1313.4	0.1	1313.5	0.9
	M6	1476.5	18.4	1476.4	19.0
	M7	1638.5	28.8	1638.4	26.7
	M8	1800.8	19.4	1800.6	20.8
	M9	1962.8	16.6	1962.8	20.0
Glucosylated *N*-glycan					
	GlcM9	2125.1	3.2	2125.8	3.9
	Glc2M9	2286.8	0.5	2286.8	0.5
	Glc3M9	2448.9	2.0	2449.1	1.8
Fucosylated *N*-glycan					
	M3F	1135.2	3.1	1135.4	0.3
N-Acetylglucosaminylated *N*-glycan					
	GNM3	1193.2	0.2		-
	GNM5	1517.1	4.3	1517.6	2.1
	GNM3F	1339.3	1.1	1338.8	1.6
	GNM5F	1662.9	0.4	1662.6	0.5
	GN2M3F	1542.0	0.8	1542.4	0.7
Total Mannose-type structure			84.4		88.7
Total Glucosylated *N*-glycan			5.7		6.2
N-Acetylglucosaminylated *N*-glycan			6.8		4.9

The relative ratio of the structures was calculated on the basis of the peak area as determined by LC-MS/MS analysis. The observed mass of the structure in LC-MS/MS analysis was shown.
